# The brain network underlying social participation: a multimodal, data-driven investigation

**DOI:** 10.1007/s11682-026-01165-3

**Published:** 2026-05-28

**Authors:** Mia G. Casburn, Aodán Laighneach, Evie Doherty, Fergus Quilligan, Andrea Fernandes, Emma Corley, Gary Donohoe, Pilib Ó Broin, Derek W. Morris, Dara M. Cannon

**Affiliations:** 1 Center for Neuroimaging, Cognition and Genomics (NICOG), Galway Neuroscience Center, University of Galway, Galway, Ireland; 2 Clinical Neuroimaging Laboratory, Galway Neuroscience Center, School of Medicine, College of Medicine, Nursing and Health Sciences, University of Galway, Galway, Ireland; 3 School of Biological and Chemical Sciences, University of Galway, Galway, Ireland; 4 School of Psychology, University of Galway, Galway, Ireland; 5 School of Mathematical & Statistical Sciences, University of Galway, Galway, Ireland

**Keywords:** Social participation, Data-Driven Analysis, Social Brain, Multimodal neuroimaging, UK biobank

## Abstract

Identifying brain phenotypes influencing social participation may help understand social deficits in psychiatric disorders. Previous research shows methodological inconsistencies, lacking consensus on which brain regions are crucial. Data-driven variable selection may overcome this, facilitating unbiased replication and discovery of social brain regions. We compare data-driven selection to literature-identification of brain regions in explaining social participation variation. In 37,576 UK Biobank participants (mean age 65 ± 8, 53% female) with structural and functional neuroimaging data, social participation (range 0–10) was derived by combining leisure activity participation and friend/family visits. First, literature review identified a subset of brain regions previously associated with social measures. Secondly, recursive feature elimination selected a subset of imaging-derived phenotypes in 25% (*n* = 9,394) of the sample. Hierarchical regression in the remaining 75% (*n* = 28,152) compared whether data-selected or literature-identified brain phenotype-sets explained more variance in social participation. Individual p-values were corrected for multiple comparisons using the false discovery rate. Recursive feature elimination selected 198 imaging-derived phenotypes. Data-selected imaging-derived-phenotypes explained more variance in social participation (1.31%) than literature-identified (0.84%, F = 3.17, *p* < 0.0001). Seventeen imaging-derived-phenotypes were associated with social participation including mid-posterior-cingulate, inferior-frontal/orbital and insular thickness, and functional connectivity between pericentral with medial frontoparietal and cerebellar networks. Multi-modal brain imaging-derived phenotypes can predict small but significant variation in social participation. We confirmed previously identified social brain associations of pericentral and medial frontoparietal, and orbital regions while also implicating novel relationships with the insula, acoustic radiation, and lateral frontoparietal networks. This highlights the value of data-driven approaches in solidifying social brain regional involvement, outperforming literature-based methods, and revealing previously undetected relationships.

**Supplementary Information:**

The online version contains supplementary material available at 10.1007/s11682-026-01165-3.

## Introduction

Social participation (SP) is associated with better quality of life (Lestari et al., 2021), physical (Leone & Hessel, 2016), and mental health outcomes (Chiao et al., 2011; Santini et al., 2020). Accordingly, social isolation is a risk factor for mortality (Holt-Lunstad et al., 2010). Individuals with psychotic disorders experience persistent severe deficits in social functioning and social withdrawal (Chang et al., 2018; Harvey et al., 2007; Hodgekins et al., 2015; Isele et al., 1985; Wiersma et al., 2000). These deficits can affect other functional markers, with poorer social functioning associated with poorer outcomes overall (Albert et al., 2011; Velthorst et al., 2017). Therefore, a strong understanding of the neurobiology of social phenotypes such as SP is imperative to facilitate understanding of social deficits.

An objective measure of SP refers to an individual physically attending/taking part in social activities (Granlund, 2013; Hoffmann et al., 2023). Variability in brain structure and function correlates with various objective measures of social phenotypes such as social network size (Bickart et al., 2011) social engagement (James et al., 2012), social activity participation (Kieckhaefer et al., 2023) frequency of friend/family visits (Anatürk et al., 2021) and online social networks (Kanai et al., 2012). The social brain hypothesis posits that social species adapted to the increased cognitive demands of larger social groups through an increase in grey-matter-volume (GMV) (Dunbar, 1998). Theories propose a “social brain network” specializing in processing socially relevant stimuli (Oesch, 2024) that could potentially explain within-species variability in human social tendencies (Bickart et al., 2011, 2012). Notably, social deficits associated with disorders such as psychosis and autism spectrum disorder may stem from aberrant functioning of this social brain network (Burns, 2006; Kennedy & Adolphs, 2012; Oesch, 2024).

However, defining the social brain network has proven challenging. For instance, while some whole-brain studies associate the entorhinal cortex (Blumen & Verghese, 2019; Kanai et al., 2012) and hippocampus (Bittner et al., 2019; Blumen & Verghese, 2019) with social phenotypes, others observe no significant relationships (Anatürk et al., 2021; Kwak et al., 2018; Lin et al., 2020; Noonan et al., 2018; Veerareddy et al., 2023). Discrepancies likely stem from variability in social parameters, methodologies or analytical approaches. Many studies hypothesize brain or seed regions which may overlook pivotal brain regions and over-represent others, introducing potential bias. A multimodal approach (Anatürk et al., 2021; Kieckhaefer et al., 2023; Noonan et al., 2018) achieves an integrated structural and functional interpretation, increasing sensitivity by identifying consistent effects across modalities and facilitating parallel conclusions into the anatomical and functional correlates of social phenotypes. Although multimodal approaches can be complex and highly computational, such methods can be made more feasible when combined with data-driven techniques.

Accordingly, data-driven approaches such as that of Lin et al. (2020) facilitate unbiased replication and discovery of reliable social brain components by establishing associations solely on existing patterns in a dataset without the constraints of prior knowledge or expectations (Maass et al., 2018). Recursive feature elimination (RFE) is a supervised feature selection method that assesses a model using the total set of candidate predictors while iteratively eliminating those that contribute the least. At each iteration, and as features are eliminated, model accuracy is evaluated and optimized until a subset that best explains the outcome is established. RFE has been shown to be efficient and largely resistant to overfitting (Guyon et al., 2002; Thakkar & Lohiya, 2021; Thanh Nhu et al., 2022). Moreover, RFE with bootstrapping and cross-validation facilitates the detection of stable relationships across multiple subsamples, and it is particularly appropriate for the UK Biobank (UKB) where thousands of Imaging Derived Phenotypes (IDPs) are available.

There is considerable disagreement about which brain regions are most critical for social functioning. Existing studies often emphasize isolated brain regions over a whole-brain, multimodal perspective, exhibiting extremely variable methodologies. This paired with hypothesis-driven approaches and inconsistent social phenotypes has produced heterogenous findings. Therefore, this investigation applies a novel multimodal, data-driven approach incorporating RFE with cross-validation to identify a subset of IDPs associated with SP in the UKB dataset. RFE is applied to multimodal measurements of cortical and subcortical structure, microstructural white matter organization, and functional connectivity (FC). We also compare these data-selected IDPs to a literature-identified IDP subset in an independent sample to establish whether a data-driven approach facilitates a better explanation of SP variance than existing literature. This method investigates the combined variance of associated regions, offering an advantage over region-specific values often reported in previous studies. Additionally, it allows for comprehensive insights into the social brain, facilitating the replication and discovery of associated IDPs across imaging modalities.

## Methods

### Study design

Neuroimaging data was available for 46,812 individuals in the UKB (2023 release). Participants were excluded if they were: missing SP data, non-Caucasian, diagnosed with psychosis, or had neurodegenerative/demyelinating/neoplastic brain diseases. Participants missing T2 FLAIR for Freesurfer processing (Fischl, 2012) were removed (Smith et al., 2024). The final sample consisted of 37,576 participants. To compare the data-selected to literature-identified IDPs, RFE was performed in 25% of the population (*n* = 9,394), with selected IDPs assessed for their association with SP independently in the remaining 75% (*n* = 28,152).

### Social phenotype

SP was a composite score created from leisure/social activities [Data-Field 6160] https://biobank.ndph.ox.ac.uk/showcase and frequency of visiting friends/family [Data-Field 1031] https://biobank.ndph.ox.ac.uk/showcase forming an objective measure of social participation. Scoring ranged from 0 to 10, with higher scoring indicating better SP (See Supplementary Methods).

### Imaging acquisition & processing

This study used processed brain IDPs derived from the workflow established and applied on behalf of the UKB (Alfaro-Almagro et al., 2018; Smith et al., 2024). Identical scanners (Siemens Skyra 3 T software VD13) and procedures (Miller et al., 2016; Smith et al., 2024) were used across all three imaging sites. We used IDPs derived from T1-weighted MPRAGE, diffusion-weighted spin-echo planar imaging (dwMRI) and resting-state functional (rsfMRI) echo-echo planar imaging. These included measurements of cortical volume, thickness, and area, subcortical volume and intensity, white-matter fractional anisotropy (FA), and rsfMRI FC measures.

### Literature-based feature identification

A search strategy applied in PubMed and Scopus, (supplemented by Google Scholar and relevant reference lists) sourced articles investigating the association between IDPs (T1, dwMRI or rsfMRI) and objective social phenotypes, defined as those minimizing the subjective opinions/feelings of participants towards their social behaviors. Inclusion criteria for studies were (1) an objective social phenotype, and (2) a non-psychosis population. Studies were excluded if they did not use a whole-brain approach or IDP measurements corresponding to UKB variables. Only results derived from whole-brain analyses were considered in mixed-methods studies. If a region mapped to multiple UKB IDPs, all corresponding IDPS were selected. Only regions associated with a social phenotype at *p* < 0.05 were included.

### Data-driven feature selection

 All IDPs were demeaned and scaled (Guyon & Elisseef, 2003). Highly correlated (> 0.80) IDP pairs were averaged (Gurholt et al., 2024) (Table S2) and separate RFEs were run for each measurement type. For measurements containing many IDPs (e.g. cortical volume IDPs *n* = 148), columns were randomized and multiple RFEs were run. RFE analyses were conducted in the 25% test sample. A 10-fold cross-validation procedure (5 repeats) and bootstrapping loop of 100 was employed to avoid over-fitting and ensure selection accuracy (Guyon & Elisseef, 2003; Lee et al., 2022). RFE evaluated subset sizes from 1-total number of input variables, selecting the highest-performing subset of IDPs. One feature was eliminated at each RFE iteration (Guyon & Elisseef, 2003). Variables selected in more than half of the bootstraps (> 50 times) were deemed stable SP predictors.

### Statistical analyses

Hierarchical linear regressions tested the relationship between each measurement-type IDP group (T1-, diffusion- and rsfMRI-derived IDP sets) and SP. The base model included covariates (age, sex, years of education, head size, social living situation and chronic illness/disability) and additional variance explained by adding either literature-identified or RFE-selected IDPs was estimated. A final regression compared RFE-selected to literature-identified IDPs across modalities, with ANOVA determining if models significantly differed. Using only overlapping measurement types (excluding cortical thickness, area and subcortical intensity), a sub-analysis assessed whether RFE outperformed the literature-identified set. In the final regression model, individual p-values were FDR-corrected using q-values (threshold q < 0.05) (Storey, 2002). Analyses were conducted in R Studio (V4.3.1) using the caret (Kuhn, 2008), q-value (Storey et al., 2023), and dplyr (Wickham et al., 2022) packages.

## Results

### Literature-based feature identification

The cortical and subcortical gray matter literature search identified fifteen studies. Among whole-brain studies examining social phenotypes (*n* = 12), the majority (*n* = 11) implicated volumes of frontal, temporal and limbic cortices, particularly pre-frontal, orbitofrontal cortex (OFC), and parahippocampal/entorhinal regions (Table 1), with few investigating cortical thickness or area. Forty-four corresponding UKB-IDPs were available for inclusion in the final literature-identified subset (Table S3).

**Table 1:** Studies Investigating the Relationship Between Objective Social Phenotypes and T1-weighted MRI Derived Grey Matter Structural Measures

Authors	Mean age	Sample sex (M: F)	Social Metric	Imaging Measure	Relevant Covariates			Associated structure & relationship direction (- or +)
**Whole brain**								
Kieckhaefeer et al. (2023)	52	18,189: 20,512	Social leisure activity participation	GMV	Age, sex, age*sex, BMI, head size, head motion.		GMV deviations in vmPFC, para-hippocampal gyrus, middle temporal, ACC, and fusiform gyri Regions in DMN & limbic networks	
Anaturk et al. (2021)	56	3,897: 3,255	Social leisure activity participation	GMV	Age, sex, education, occupation, BMI, alcohol intake, sleep, mental health, arterial pressure people in household, head size		No associations	
			Frequency of friend/family visits				Occipital pole (+)	
Bittner et al. (2019)	67	301: 248	Social Integration Index	GMV	Age & sex		Left hippocampus (+)	
				Gyrification			Right vlPFC (+)	
James et al. (2012)	65	348: 0	Engagement in social activities	GMV	Handedness, age, race, education, hypertension, diabetes, ICV		Total GMV (+)	
Veerareddy et al. (2023)	20	93: 99	SNI	GMV	Age, sex, ICV		Left dmPFC (-)	
Kanai et al. (2012)	23	46: 79	SNI	GMD	Age, sex, ICV		Right amygdala (+)	
			Number of online friends	GMD			Left middle temporal gyrus, right posterior superior temporal sulcus, right entorhinal (+)	
Lin et al. (2020)	30	51: 41	SNI	GMV	Separate demographic analysis		No associations	
Blumen and Verghese (2019)	75	46: 40	SNI	GM covariance patterns	Age, sex, education, global health status, ICV		mPFC, lPFC, OFC, parahippocampal gyrus, hippocampus, entorhinal, amygdala, pre-cuneus, pre-central gyrus, insula, cingulate, thalamus, palladium, cerebellum & brainstem (+)	
Kwak et al. (2018)	71	26: 42	In-degree SNS	GMD	Age, sex, education, ICV		Right superior frontal gyrus Right OFC (+) Left OFC (+) [*p* = 0.055 corrected]	
			Out-degree SNS				No associations	
Bickart et al. (2011)	53	36: 22	SNI	Cortical Thickness	Age, sex, ICV		Thickness of caudal inferior temporal sulcus, superior frontal gyrus, subgenual ACC (+) [*p* < 0.01 uncorrected for multiple comparisons]	
Lewis et al. (2011)	27	19: 26	Number of social contacts in last 30 days	GMV	Age, sex		vmPFC [OFC & medial frontal gyrus] (+)	
Noonan et al. (2018)	52	7: 11	Number of social contacts in last 30 days	GMV	Age, sex, years of education, ICV, head motion		vmPFC, amygdaloid complex, temporal pole, posterior cingulate/pre-cuneus (+)	
**Region-of-Interest**								
James et al. (2012)	65	348: 0	Engagement in social activities	GMV of 20 regions	Handedness, age, race, education, hypertension, diabetes, ICV	Temporal & Occipital lobes & corpus callosum (+)		
Kanai et al. (2012)	23	46: 79	SNI	GMD of the amygdala	Age, sex, ICV	SNI: No significant associations		
			Number of online friends			Online friends: Bilateral amygdala (+)		
McGugin et al. (2024)	22	46:71	SN Size	GMV	Age, sex, ICV	No associations		
			SN Complexity			No associations		
			Online SNS			No associations		
			Real-World SNS			Central nucleus of the amygdala (+)		
Bickart et al. (2011)	53	36: 22	SNI sub-scale: Number of regular contacts	GMV of the amygdala & hippocampus	Age, sex, ICV	Contacts: left & right amygdala (+)		
			SNI sub-scale: Number of social groups			Groups: left & right amygdala and left hippocampus (+)		
Powell et al. (2012)	26	17: 23	SNQ	GMV of PFC	Sex, hemisphere volume	OFC (+)		
VonDerHeide et al. (2014)	20	0: 40	Number of social contacts in last 30 days	GMV of right ACC, left inferior temporal sulcus, superior frontal gyrus, bilateral amygdala, superior temporal sulcus, middle temporal gyrus, entorhinal, & OFC	Age, ICV	Dunbar’s number: Bilateral amygdala, left posterior superior frontal gyrus, right entorhinal, bilateral OFC (+)		
			NSSQ			Bilateral amygdala, left inferior temporal sulcus, bilateral OFC, right entorhinal (+)		
			Online: Number of Facebook friends			Bilateral amygdala, bilateral OFC, left superior frontal gyrus, left entorhinal (+)		
Noonan et al. (2018)	52	7: 11	Number of social contacts in the last 30 days	GMV of ACC	Age, sex, years of education, ICV, head motion	left ACC (+)		

***Legend***: Table indicating regions where GMV was associated with social phenotypes in previous studies. Rows are ordered by sample size from largest to smallest. Only regions associated with a social phenotype in whole-brain studies at *p* < 0.05 were identified as literature-based features. SNS Social Network Size, SNI Social Network Index, SNQ Social Network Questionnaire, NSSQ Norbeck Social Support Questionnaire, In-Degree SNS is how many people named the participant as a social contact, Out-Degree SNS is how many people the participant named as a social contact. ICV intra-cranial volume, GMV gray matter volume, ROI region of interest, OFC orbitofrontal cortex, PFC pre-frontal cortex, ACC anterior cingulate cortex

The dwMRI-derived white matter microstructural organization literature review identified four papers (Table 2), three employing a whole-brain approach. Overall, FA in five tracts was associated with social phenotypes, most consistently implicating the cingulum and the corpus callosum (Molesworth et al., 2015; Noonan et al., 2018). One study (Noonan et al., 2018) implicated the superior longitudinal/arcuate fasciculus, inferior front-occipital fasciculi, and the inferior longitudinal fasciculus. One study identified no significant associations (Anatürk et al., 2021). Twelve corresponding UKB-IDPs were available (Table S3).

**Table 2:** Studies Investigating the Relationship Between Objective Social Phenotypes and Diffusion-weighted MRI-derived White Matter Phenotypes

Authors	Mean age	Sample sex (M: F)	Social Metric	Imaging Measure	Relevant Covariates	Associated structure & relationship direction (- or +)
**Whole brain**						
Anaturk et al. (2021)	56	3,897 : 3,255	Social leisure activity participation	FA MD	Age, sex, education, occupation, BMI, alcohol intake, sleep, mental health, arterial pressure people in household, head size	No associations
			Frequency of friend/family visits	FA MD		No associations
Molesworth et al. (2015)	41	78 : 77	SNI	RD	Age, sex, education, central adiposity	Whole-brain RD (-)
				FA		Whole-brain FA (+) FA in the dmPFC & posterior genu of the corpus callosum (+) FA between the superior/middle frontal gyrus, left cingulum & superior infundibulum [connections between right and left dlPFC] (+)
Noonan et al. (2018)	52	9: 11	Number of social contacts in last 30 days	FA	Age, sex, years of education, ICV, head motion	FA in the right cingulum, arcuate fasciculus, inferior front-occipital fascicle [extreme capsule], inferior longitudinal fasciculus & corpus callosum (+)
**Region-of-Interest**						
Hampton et al. (2016)	21	0 : 35	NSSQ	FA between amygdala ATL	Age	FA in tracts connecting the amygdala to the ATL accounted for 33% of variability in NSSQ
				FA between amygdala and OFC		FA in tracts connecting the amygdala to the OFC accounted for 10% of variability in NSSQ Age, amygdala-ATL FA and amygdala-OFC FA explained 69% of variability in NSSQ

***Legend***: Table indicating white-matter tracts where FA was associated with social phenotypes. Rows are ordered by sample size from largest to smallest. Only tracts associated with a social phenotype in whole-brain studies at *p* < 0.05 were used for literature-based feature identification. SNS Social Network Size, SNI Social Network Index, NSSQ Norbeck Social Support Questionnaire. ROI region-of-interest, FA fractional anisotropy, MD mean diffusivity, RD radial diffusivity, OFC orbitofrontal cortex, PFC pre-frontal cortex, ATL anterior temporal lobe

Finally, eleven studies (Table 3) investigating FC and social phenotypes included three whole-brain studies primarily implicating within-network FC of the pericentral and lateral/medial occipital networks, between-network FC of the pericentral with medial and lateral frontoparietal, occipital and cerebellar networks (Anatürk et al., 2021; Kieckhaefer et al., 2023; Pillemer et al., 2017). These connections correspond to 81 UKB-IDPs (65 between- and 16 within-network, Table S4). The remaining three whole-brain studies highlighted FC in frontotemporal and amygdala regions, did not implicate specific resting-state networks (Bang et al., 2019; Joo et al., 2017) or used a measure not available in the UKB (Wang et al., 2022).

**Table 3:** Studies Investigating the Relationship Between Objective Social Phenotypes and rsfMRI-Derived Functional Connectivity Phenotypes

Authors	Mean age	Sample sex (M: F)	Social Metric	Imaging Measure	Relevant Covariates	Associated structure & relationship direction (- or +)
**Whole brain**						
Kieckhaefeer et al. (2023)	52	18,189: 20,512	Social leisure activity participation	FC	Age, sex, age*sex, BMI, head size, head motion.	Inter-network FC of the default mode network, limbic and frontoparietal networks with the somatomotor network (+)
Anaturk et al. (2021)	56	3,897: 3,255	Social leisure activity participation	FC	Age, sex, education, occupation, BMI, alcohol intake, sleep, mental health, arterial pressure people in household, head size	FC between sensorimotor and lateral visual RSNs (+) FC between sensorimotor and cerebellar RSNs (+)
			Frequency of friend/family visits			No association
Wang et al. (2022)	22	92: 138	General social acquaintances	fALFF	Age, sex, general intelligence, head motion	fALFF in the vmPFC, right superior temporal gyrus, OFC and pre-cuneus (all +)
Bang et al. (2019)	73	25: 36	In-degree SNS	FC	Age, sex, years of education, smoking & drinking status, BMI, metabolic disorders	No associations
			Out-degree SNS			FC between dorsal ACC/anterior PFC & temporal pole/fusiform gyrus (+)
Pillemer et al. (2017)	72	12: 16	SNI	FC	Not available	FC within sensorimotor network (primary motor, somatosensory, dmPFC), visual network (primary & secondary visual cortex), vestibular/insular network (insula and vmPFC), and left frontoparietal (dmPFC, vmPFC, posterior cingulate, precuneus) (all +)
**Region-of-Interest**						
Mori and Haruno (2022)	22	149: 73	Online reply Network: number of accounts that responded to participant’s posts	FC between 36 social brain atlas regions	Age, sex, alcohol use, nicotine dependence, socio-economic status	FC between left IFG & dmPFC, bilateral temporal pole, left TPJ, left middle temporal gyrus (+) FC between frontal pole & left TPJ, bilateral temporal pole, & dmPFC (+) FC between rACC & posterior cingulate, left temporal pole, right TPJ and right middle temporal gyrus (+)
			Online follow Network: Number of followers			No associations found with Follow Network
Pruitt et al. (2024)	71	28: 84	Lubben Social Network Scale	FC in default mode & salience networks	Age, sex	FC within the salience network (+)
Bickart et al. (2012)	22	44: 45	SNI	FC in lateral OFC, vmPFC, ACC, amygdala	ICV	FC between medial amygdala & vmPFC (+) FC between ventrolateral amygdala & superior temporal sulcus, rostral & caudal hippocampus, OFC & fusiform gyrus (+)
Joo et al. (2017)	71	22: 42	Social network size	FC	Age, sex, Mini-Mental State examination score	FC between temporal lobe (amygdala, fusiform, middle temporal) & occipital (calcarine fissure, lingual, inferior occipital gyrus) (+)
			Social network embedeness			FC between right temporal lobe (inferior frontal, insula) & occipital (cuneus, superior occipital, middle occipital gyrus) (+)
Zou et al. (2016)	31	16: 15	SNI	FC between amygdala and OFC	Age, sex, education level, smoking index	FC between the right amygdala & left medial OFC (+) FC between the left amygdala & left medial OFC (+)
Noonan et al. (2018)	52	8: 11	Number of social contacts in last 30 days	FC of regions in the DMN	Age, sex, years of education, head motion, ICV	FC between dlPFC/ACC & anterior DMN (vmPFC, cingulate, temporal pole, thalamus, hippocampus) (+)

***Legend***: Table indicating regions where FC was associated with social phenotypes. Rows are ordered by sample size from largest to smallest. Only regions associated with a social phenotype in whole-brain studies at *p* < 0.05 were identified as part of the literature-based feature set. SNS Social Network Size, SNI Social Network Index, In-Degree SNS is how many people named the participant as a social contact, Out-Degree SNS is how many people the participant named as a social contact. ROI region-of-interest, FC functional connectivity, fALFF fractional amplitude of low-frequency fluctuations, RSN resting state network, OFC orbitofrontal cortex, PFC pre-frontal cortex, ACC anterior cingulate cortex, TPJ temporo-parietal junction, IFG inferior frontal gyrus, DMN default mode network

### Data-driven participants & feature selection

The total sample included 37,576 participants without psychosis, of White British/Caucasian ancestry with an age range of 45–83 (mean age 64.5 ± 7.67, 53.3% female), a mean of 15.61 years of education (=/- 4.54) and mean SP score of 4.48 (+/- 1.50). Of the total sample, 37,058 had data on chronic illness/disability and social living situation, 19.2% (*n* = 7,120) lived alone, 76.5% (*n* = 28,358) lived with a partner/spouse and 4.27% (*n* = 1,580) lived with other people including friends, parents etc., while 26.22% (*n* = 9,718) reported living with a disability, illness or infirmity. This sample was split, with 25% (*n* = 9,394, 53.1% female, mean age 64.47+/−7.64) used for feature selection and 75% (*n* = 28,152, 52% female, mean age 64.47+/−7.68) for regression analysis.

Of the 442 cortical IDPs, RFE selected 43 (29.1%) mean thickness, 29 (19.6%) volume, and 6 (4.1%) area. Regions selected across all three measurement-types included the central and pre-central sulci, and orbital gyri and sulci (Table S6). Of the 31 subcortical volumes included, RFE selected 11 (35.5%, Table S7) as optimally predicting SP. All 31 subcortical intensity IDPs were selected.

RFE selected 20 of 21 (95.2%) probabilistic tractography (PT) derived and 9 of 34 (26.4%) Tract Based Spatial Statistics (TBSS) -derived white matter tracts. Fractional anisotropy (FA) of the middle cerebellar peduncle, superior longitudinal fasciculus (bilaterally) and the cingulum were selected across both PT and TBSS IDPs (Table S8).

Finally, of the 210 rsfMRI IDPs, RFE selected 49 (23.3%) including within- and between-network edges. Nine reflected within-network FC in the medial (4) and lateral frontoparietal (2), pericentral (2), and medial occipital (1) networks. Between-network edges containing nodes of the medial frontoparietal (24 of the 49 selected edges) and pericentral (23) networks were most prominent. Between-network connectivity of the medial frontoparietal with the lateral frontoparietal and pericentral networks showed the highest frequency (7 edges each) (Table S9).

### Overlap between literature-identified and data-selected features

Across all measurement types, 42 literature-selected IDPs were also identified as important in the RFE analysis, representing 35.6% of the RFE-selected variables. Functional connectivity showed the most consistent overlap between literature- and data-selected subsets, with 19 IDPs (38.8% of the data-selected IDPs), followed by 11 cortical GMV (38.0%), 4 subcortical GMV (36.4%), and 8 FA (27.6%). The literature- and data-selected sub-sets of IDPs were carried through to the next analysis step, where regression analyses were conducted in the remaining 75% of the sample to determine whether the unique selections identified by RFE explained more SP variance.

### Data-driven feature selection outperforms literature-based feature identification

Across all modalities, data-driven feature selection outperformed literature. While age, sex, years of education, head size, chronic illness/disability and social living situation explained ~ 5.02% of the variance in SP (Table 4), data-selected T1-derived IDPs explained more variance (0.45%) than literature-identified (0.08%), as did diffusion-derived features (literature R^2^ = 0.01%, RFE R^2^ = 0.15%). Linear regression of the rsfMRI data showed the highest additional variance explained across imaging types (literature R^2^ = 0.74%, data R^2^ = 0.84%).

**Table 4:** Hierarchical Linear Regressions Comparing Associations of Literature-Identified versus Data-selected IDP Sub-sets with Social Participation

	*r* (*R*^2^)	Additional *R*^2^		*r* (*R*^2^)	Additional *R*^2^
**T1 – GMV, thickness, area, intensity**					
Base	0.05018 (5.02%)		Base	0.05018 (5.02%)	
+ Literature-Identified	0.05096 (5.10%)	+ 0.08%	+ Data-Selected	0.05469 (5.47%)	+ 0.45%
**dwMRI - FA**					
Base	0.05067 (5.07%)		Base	0.05067 (5.07%)	
+ Literature-Identified	0.0508 (5.08%)	+ 0.01%	+ Data-Selected	0.05216 (5.22%)	+ 0.15%
**rsfMRI - FC**					
/Emphasis>Base	0.05059 (5.06%)		Base	0.05059 (5.06%)	
+ Literature-Identified	0.05796 (5.80%)	+ 0.74%	+ Data-Selected	0.05897 (5.90%)	+ 0.84%
**All IDPs**					
Base	0.05063 (5.06%)		Base	0.05063 (5.06%)	
+ Literature-Identified	0.05894 (5.89%)	+ 0.83%	+ Data-Selected	0.06372 (6.37%)	+ 1.31%

***Legend***: Base: Covariates age, sex, years of education, chronic illness/disability, social living situation, and head size. Numbers indicate r and R^2^ values of each model and “Additional R^2”^ indicates the additional variance explained. Variance explained for covariates is slightly different for each as some participants did not have data for all imaging modalities. IDPs imaging derived phenotypes, GMV grey matter volume, dMRI diffusion-weighted MRI, FC functional connectivity

Combined across modalities, data-selected IDPs explained significantly more SP variance (1.31%) than literature-identified (0.84%, F = 3.17, *p* < 0.0001). Across all measurement types, 17 of 198 data-selected IDPs were associated with SP after FDR correction (Table 5) including thickness of the left mid-posterior cingulate, frontal-orbital gyrus, and superior-circular insular sulcus. Additionally, connectivity between the medial frontoparietal with the pericentral, lateral frontoparietal and medial occipital networks, and FC of the cerebellar network with the pericentral and lateral occipital. Finally, FA values in the left acoustic radiation, right cerebral peduncle and intensity of the right amygdala and average putamen survived FDR correction. No cortical area IDPs survived correction. The sub-analysis removing previously uninvestigated measurement types (thickness, area and intensity) showed that the data-selected sub-set still explained more variance than literature (additional R^2^ = + 1.09% and + 0.84%, respectively). Finally, we tested if weighting for lower frequency SP values at the distribution tails improved variance explained using the covariates, however this resulted in overfitting (R²=25.30% without cross-validation; 10-fold cross-validated R²=3.65%).

**Table 5:** Data-Selected IDPs Significantly Associated with Social Participation in the Final Multimodal Linear Regression

Variable	Standardized Coefficient	T Value	Unadjusted *P*-value	FDR Adjusted Q-value
**Covariates**				
Age	0.035	17.23	< 2 × 10^− 16^	**4.51 × 10** ^**− 64**^
Sex (0 = male, 1 = female)	0.52	17.05	< 2 × 10^− 16^	**4.77 × 10** ^**− 63**^
Years of Education	0.02	10.63	< 2 × 10^− 16^	**9.99 × 10** ^**− 25**^
Social Living Situation (compared to living alone)				
Living with others (not partner)	−0.147	−3.01	0.003	**0.003**
Living with partner	−0.143	−6.13	9.10 × 10–10	**9.10 x x 10** ^**− 10**^
Chronic Illness/Disability	−0.072	−3.46	0.00054	**0.0008**
**Subcortical Volume**				
L Cerebellum White Matter	0.033	2.26	0.02	0.09
**Subcortical Intensity**				
R Amygdala	−0.040	−2.80	0.005	**0.041**
BA Putamen	0.049	2.70	0.007	**0.041**
L Pallidum	−0.038	−2.50	0.013	0.084
R Pallidum	0.037	2.46	0.014	0.065
BA Ventral Diencephalon	−0.040	−2.42	0.015	0.069
R Cerebellum White Matter	0.027	2.02	0.042	0.117
**Cortical Volume**				
L Triangular part of the Inferior Frontal Gyrus	−0.030	−2.61	0.009	**0.048**
R Sub-Callosal Gyrus	0.026	2.27	0.023	0.090
R Superior Temporal Sulcus	0.031	2.17	0.03	0.099
**Cortical Thickness**				
L Inferior Frontal Orbital Gyrus	−0.034	−2.77	0.006	**0.041**
L Mid-Posterior Cingulate G + S	0.050	4.02	5.78 × 10^− 05^	**0.001**
L Superior-Circular Sulcus of the Insula	−0.040	−3.11	0.002	**0.02**
L Paracentral G + S	−0.039	−2.52	0.011	0.057
R Inferior Temporal Gyrus	0.027	2.19	0.023	0.10
R Middle Temporal Gyrus	−0.031	−2.12	0.034	0.106
**Cortical Area**				
L H-Shaped Orbital Sulcus	−0.054	−2.19	0.028	0.10
**TBSS mean FA**				
R Cerebral Peduncle	0.04	2.76	0.006	**0.041**
**PT weighted-mean FA**				
L Acoustic Radiation	−0.034	−2.68	0.007	**0.041**
R Medial Lemniscus	−0.027	−2.32	0.020	0.084
R Cingulum (para-hippocampal segment)	−0.230	−1.99	0.046	0.122
**Functional Connectivity**				
** Within-Network FC**				
Medial Frontoparietal/Mid-Cingulo-Insular (Edge 67)	−0.022	−2.31	0.021	0.084
Medial Frontoparietal/Mid-Cingulo-Insular (Edge 184)	0.02	2.02	0.043	0.12
** Between-Network FC**				
Lateral Frontoparietal & R Medial Frontoparietal (Edge 199)	0.048	5.02	5.27 × 10^− 07^	**1.24 × 10** ^**− 05**^
R Lateral Frontoparietal & Medial Frontoparietal/Mid-Cingulo-Insular (Edge 71)	0.040	4.00	6.25 × 10^− 05^	**0.001**
L Lateral Frontoparietal & Medial Frontoparietal (Edge 21)	0.02	1.98	0.046	0.122
Medial Frontoparietal & Pericentral (Edge 56)	0.03	2.75	0.006	**0.041**
Medial Frontoparietal & Pericentral (Edge 182)	0.03	2.70	0.007	**0.041**
Medial Frontoparietal & Pericentral (Edge 43)	0.020	2.18	0.029	0.10
Lateral Frontoparietal & Pericentral (Edge 136)	0.019	2.09	0.037	0.11
Lateral Frontoparietal & Pericentral (Edge 60)	−0.02	−2.06	0.039	0.11
Lateral Frontoparietal & Pericentral (Edge 9)	−0.02	−2.12	0.034	0.11
Lateral Frontoparietal & Pericentral (Edge 115)	−0.019	−2.03	0.043	0.12
Pericentral & Cerebellar (Edge 103)	−0.04	−3.16	0.002	**0.019**
Pericentral & Basal Ganglia (Edge 148)	−0.030	−2.16	0.031	0.10
Pericentral & Medial Occipital (Edge 6)	0.03	3.02	0.003	**0.024**
Medial Frontoparietal & Medial Occipital (Edge 4)	0.032	3.37	0.001	**0.009**
Lateral Occipital & Cerebellar (Edge 93)	−0.027	−2.72	0.007	**0.041**
Lateral Occipital & Lateral Frontoparietal (Edge 30)	0.027	2.58	0.01	**0.049**

## Discussion

Social participation is associated with variations in multi-modal brain metrics including thickness of the mid-posterior cingulate gyrus and sulcus, orbital gyrus, and the superior-circular insular sulcus, functional connectivity of the medial frontoparietal network (MFP) to the lateral frontoparietal, pericentral and medial occipital networks and FA values in the cerebral peduncle and acoustic radiation. Data-driven feature selection out-performed literature-identified when applied to multimodal neuroimaging datasets, implicating IDPs that both replicate and expand previous knowledge.

## Findings & literature overlap

We demonstrate that data-driven methods such as RFE, when combined with large-cohort data, offer enhanced sensitivity and added value for detecting brain-based associations with complex behavioral phenotypes. RFE-selected IDPs explained significantly more SP variance across all modalities compared to literature, and identified IDPs previously associated with social phenotypes, namely, connectivity between the pericentral and cerebellar networks (Anatürk et al., 2021), and (although previously detected as GMV) the thickness of the left mid-posterior cingulate gyrus and sulcus (Blumen & Verghese, 2019; Noonan et al., 2018). Two IDPs representing FC between the pericentral and medial frontoparietal networks were identified, marking this as a core brain characteristic associated with SP (Kieckhaefer et al., 2023).

Notably, both the thickness and volume of the left inferior frontal gyrus were associated with social participation, replicating previous findings implicating this region in the social brain (Blumen & Verghese, 2019; Kwak et al., 2018; Lewis et al., 2011). In particular, the orbital inferior frontal gyrus is one of the most widely replicated associations across both whole-brain (Blumen & Verghese, 2019; Lewis et al., 2011; Noonan et al., 2018) and ROI (Powell et al., 2012; Von Der Heide et al., 2014) studies and our results further solidify this region as implicated in social behavior.

Our findings of small but significant effects indicate that the brain may not be as strongly associated with social phenotypes as previously thought. Lin et al. (2020) found no significant associations, arguing that inflated and biased effect sizes may have resulted in prior false positives. Although their findings are polarized compared to previous literature, our highly statistically powered results suggest a middle ground, revealing small but significant associations with seven brain phenotypic measures. For studies interested in solidifying brain phenotypes important for social behavior, data-driven methods combined with large sample sizes evidently provide an open way to approach research questions without pre-defined hypotheses. Covariates accounted for a substantial proportion of social participation variance. Lower SP was observed in younger individuals, those with a chronic illness/disability, individuals living with a partner, and men, whereas higher SP was observed in women, individuals with higher education, and those living alone. This underscores the importance of accounting for key demographics when examining neural correlates of a complex behavioral phenotypes such as SP.

## The amygdala and newly implicated regions

We did not observe several previously reported associations, including the volume of the amygdala. Regarding the amygdala, our review showed differing associations across ROI and whole-brain studies. Amygdala GMV was associated with social phenotypes in 3 of 6 (50%) hypothesis-driven studies (Bickart et al., 2011; McGugin et al., 2024; Von Der Heide et al., 2014), but only 2 of 9 (22.22%) whole-brain analyses (Blumen & Verghese, 2019; Noonan et al., 2018), suggesting that hypothesis-driven methods potentially result in a less consistent literature base as a whole (Lin et al., 2020). Data-driven whole-brain approaches reduce such risks, providing impartial, cohesive identification and confirmation of key social brain regions such as the amygdala detected as significantly associated with SP herein (sub-cortical intensity of the right amygdala, Table 5).

Data-driven discovery identified novel associations, including the superior-circular insular sulcus, functional connectivity between medial and lateral frontoparietal networks and between medial frontoparietal and occipital networks and FA in the acoustic radiation. Notably, one edge linking the lateral frontoparietal with a combined medial frontoparietal (DMN) and mid-cingulo-insular (salience) component was associated with SP, consistent with the triple network model of resting state networks. Interestingly, these findings suggest the combined involvement of affective, control and salience/limbic systems in supporting social behavior.

## Overlap with social cognition

Many IDPs identified in our analysis overlap with regions implicated in social cognition. Kennedy and Adolphs (2012) view behaviors such as social participation as enabled by bidirectional relationships with social cognition and social functioning, further underpinned by coordination across networks combining different aspects of social processes (e.g. social perception, sensory integration, emotion recognition, etc.).

Associations with SP were shown with connectivity and thickness of the mid-posterior cingulate, a core MFP/DMN region involved in foundational socio-cognitive processes such as emotion processing (Maddock et al., 2003). Similarly, SP was associated with MFP connectivity patterns and thickness of pre-frontal and OFC sub-regions which support social perception (Van Overwalle, 2009). Interestingly, both regions have been linked to theory-of-mind (Rilling et al., 2004; Van Overwalle, 2009) which is unsurprising given the foundational role of theory-of-mind abilities in social communication (Burns, 2006; Stiller & Dunbur, 2007).

Notably, Powell et al. (2012) showed that socio-cognitive abilities mediate associations of OFC volume with the SNI, highlighting cognition as important in linking the brain with social behaviors. The overlap of our results with socio-cognitive brain regions warrants further investigation into the role that cognition plays in brain-behavior associations.

### Limitations

The cross-sectional nature of this study means that no causal or directional inferences could be made. We aimed to understand the brain phenotypes underlying SP, but it is equally likely that varying levels of social participation alter brain characteristics (Anatürk et al., 2021; Düzel et al., 2019; James et al., 2012). Future longitudinal or mendelian randomization designs may assist in establishing the directional mechanisms of SP and its relationship with neurobiology.

Finally, an objective SP measure was core to this investigation, however, in the UKB, the data available for this was limited, restricting our ability to capture the multi-faceted nature of SP and potentially missing other valuable drivers such as social contacts and informal social activity participation. Moreover, the age range in the UKB is limited to primarily older (45–83 years) and retired individuals reducing generalizability to other samples and limiting our ability to consider other potentially relevant factors to SP such as socio-economic status or employment.

## Conclusions

Our data-driven approach identified pericentral medial frontoparietal, inferior frontal/orbital, and limbic regions as important for SP using an unbiased multimodal imaging approach. The identification of MFP/DMN regions across modalities highlights that adopting a network-based, multimodal perspective may enhance the neurobiological understanding of the social brain. Feature selection used in parallel with previous knowledge is advantageous for concurrently solidifying previous theories, identifying potentially inflated associations, and uncovering novel involvement. Overall, structural and functional brain imaging-derived-phenotypes explained 1.31% of the social participation variance, highlighting the small but significant contribution of neurobiology to explaining complex, multi-faceted social phenotypes.

## Supplementary Information

Below is the link to the electronic supplementary material.

Supplementary Material 1

## Data Availability

The dataset used is not publicly available as Uk Biobank require a data access application however, information on how to access the data can be found here https://www.ukbiobank.ac.uk/use-our-data/apply-for-access/.

## Code Availability

Available upon request.
