# Supplementary material for: The brain network underlying social participation: a multimodal, data-driven investigation
Source: Brain Imaging Behav. 2026 May 28;20(3):96. doi: 10.1007/s11682-026-01165-3 (PMC13219186; doi:10.1007/s11682-026-01165-3)
Supplement: Supplementary file 1 — Supplementary Material 1 [file 11682_2026_1165_MOESM1_ESM.docx]

## **Supplementary Methods**

***Sample:*** The UK Biobank is a large prospective cohort study containing data pertaining to cognitive, lifestyle, physical health, medical records and biological samples for 502,355 participants in the United Kingdom. The starting sample for this study was 46,851 individuals with brain imaging. The results of this study feed into a larger investigation incorporating imaging with genetics in a psychosis population, therefore anyone with non-Caucasian ethnic ancestry (n = 7,224) and defined as an individual with psychosis (n = 201) were excluded from the sample. Adhering to the recommendations of the UK Biobank Imaging Documentation (Smith et al., 2024), anyone without T2 FLAIR used in addition to T1 for Freesurfer processing were removed (n = 1,117) to avoid bias. Finally, any participants with diagnoses such as neurodegenerative (G30-G32), demyelinating (G35-G37) and neoplastic (C71) that could significantly alter brain structure and function (n = 274) and without SP data (n = 459) were excluded, resulting in a final total sample of 37,576. The age covariate matched the image acquisition visit (instance 2) and years of education was derived based on educational attainment (2006-2010) according to the procedure by Okbay et al. (2022). The social living situation covariate was derived based on the variable “How are the other people who live with you related to you?” [Data field 6141] where individuals who live alone were coded as 0, individuals who live with others but not a partner (eg. parents, grandparents) were coded as 1 and individuals who live with a partner coded as 2.

***Social Phenotype:*** For leisure/social activities participants were asked “Which of the following do you attend once a week or more often?(You can select more than one)” with the options sports club/gym, pub/social club, religious group, adult education class, other, or none of the above. Participants were scored between 0 (no activity participation) and 5 (weekly participation in all activities). For frequency of visits, participants ranked how often they visit family/friends along a 6-point scale from 0 indicating (Never/Almost never) to 5 (Daily/Almost Daily). A score was then created which combined these two variables, where an individual's social participation consisted of a sum of their leisure activities and their frequency of visits responses. For example, someone who participated in 2 weekly activities and saw their friends and family daily was given a social participation score of 7 [2+5=7]. Mean SP score was 4.48 with 67.9% of participants scoring between 4-6, 24.3% between 0-3 and 7.83% between 7-10.

***Literature-driven feature selection:*** Three sets of keywords were established to fine-tune the search specificity: Set A included neuro-imaging related search terms, Set B included various comparably defined objective social measures, and Set C narrowed the search to only healthy populations. These sets were combined by using the Boolean operator “AND” and applied to titles, keywords and abstracts to ensure that only articles pertaining to at least one keyword from each set were shown such as “grey matter volume AND social participation AND healthy controls”. Objective social phenotypes were defined as those that minimise the subjective opinions/ feelings of participants towards their social behaviours. For example, social network size was considered objective as it reflects a measure that is concrete, quantifiable, and less likely to be altered by opinion than measures reflecting an individual’s perceived quality of/satisfaction with their social contacts.

***Image Acquisition***: 3D structural T1-weighted MPRAGE sequences were acquired (FOV:256 mm, slice thickness 1 mm, 1x1 mm pixels, repetition time [TR]: 2000ms, inversion time [TI]: 800ms). Diffusion-weighted spin-echo planar imaging sequences (2 mm resolution) were acquired using two b-values with 100 different diffusion-encoding directions (50 x b=1000 s/mm2 and 50 x b=2000 s/mm2, 100 diffusion-encoding directions) and multiband acceleration factor of 3 (FOV: 104x104 mm, echo time [TE]: 92ms, TR: 3600ms). Resting-state fMRI image acquisition used gradient echo-echo planar imaging (GE-EPI) with 8 multi-slice acceleration factor at 490 timepoints over 6 minutes (FOV: 88x88x64 mm, 2.4mm voxel dimensions, flip angle 52°, TE: 39 ms, TR: 0.735s). Full information on the imaging acquisition protocol and processing is described in the UKB imaging Documentation (Smith et al., 2024) and Alfaro-Almagro et al. (2018).

***Imaging-Derived Phenotypes:*** T1-structural IDPs included subcortical volumes and intensities derived within the UKB pipeline using the Freesurfer aseg tool (Fischl, 2012) and cortical volume, area and thickness measures derived using the Destrieux [Data-Field 197] (Destrieux et al., 2010) parcellation as they include both gyral and sulcal IDPs. Diffusion-based fractional anisotropy IDPs were derived using both tract-based spatial statistics (TBSS) for diffusion-tensor modelling and probabilistic tractography (PT). Finally resting-state IDPs were derived following temporal demeaning and variance normalisation where a low-dimensionality Group-PCA was applied to all images using MELODIC’s Incremental Group-PCA (MIGP) (Smith et al., 2014). Next, an ICA was applied to the MIGP output with 25 dimensions of ICA components applied. Of the 25 components, 4 were deemed artefactual resulting in 21 final components. IDPs were not normalised by head size due to the advice of the UKB Imaging Documentation stating it is only appropriate to do this with raw volume IDPs, and it would be safer to include these as confounds/covariates in later analyses when using different types of imaging measures (Smith et al., 2024). Therefore, head size scaling factor was included to account for any variability in the associations that could be confounded by variations in head size. All available IDPs in the Uk Biobank dataset can be seen at the Uk Biobank website data showcase viewer (<https://biobank.ndph.ox.ac.uk/showcase/label.cgi?id=508>).

***Resting-state fMRI network identification:*** Using the UKB online node viewer, co-ordinates at peak activation sites were manually gathered for each of the 21 components from the group-average ICA maps. These co-ordinates were each analysed using the JuBrain Anatomy Toolbox in SPM12 (Eickhoff et al., 2005) which gave a probabilistic estimate of the anatomy underlying each component. The anatomical labels assigned to each co-ordinate were then cross-referenced with several papers that provide classifications linking anatomical regions to various well-established resting-state networks (Beckmann et al., 2005; Laird et al., 2011; Smith et al., 2009; Uddin et al., 2019). This was completed by two independent researchers and a third researcher was consulted in the case of a disagreement in network classification. Each component was then named according to the anatomical nomenclature provided by Uddin et al. (2019), Bellani et al. (2020) and Robinson et al., (2009). The 21 components were classified into seven network classifications including the medial frontoparietal, mid-cingulo insular, lateral frontoparietal, occipital, pericentral (Uddin et al., 2019), cerebellar (Bellani et al., 2020) and basal ganglia networks (Robinson et al., 2009). The UKB IDPs for resting-state data consist of edges representing correlations between each of these components (Table S1).

## **Table S1.** Resting-state Nodes Peak Coordinates & Network Classifications.

| **Component/Node [Right / Left]** | **Neuroanatomy** | **MNI Coordinates (x, y, z)** | **Network** |
| --- | --- | --- | --- |
| **Node 1**  **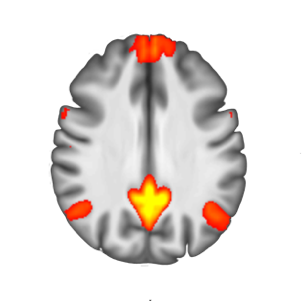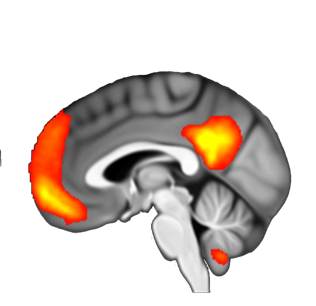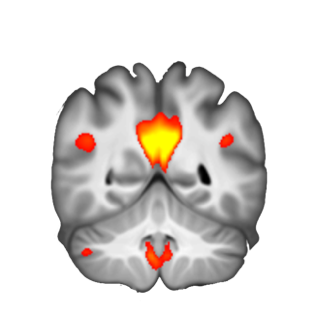** | 42% Precuneus, 40% posterior cingulate  60% Posterior Cingulate, 10% Precuneus  66% Frontal pole, 16% Frontal medial cortex | 0, -55, 27  -8, -51, 30  -2, 57, -11 | Medial Fronto-Parietal |
| **Node 2**  **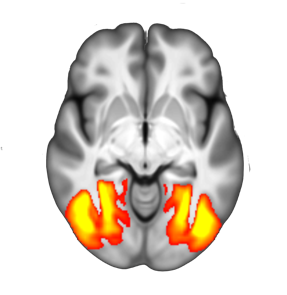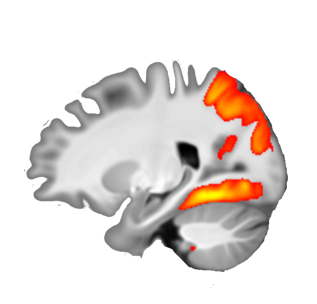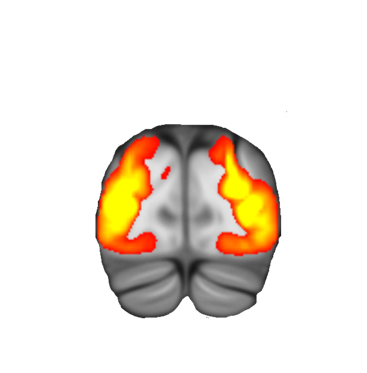** | 45% Superior Lateral Occipital Cortex  28% Inferior Lateral Occipital, 12% superior lateral occipital  51% Temporal Occipital Fusiform, 24% Lingual | -25, -78, 27  38, -78, 9  28, -53, -8 | Lateral Occipital Network |
| **Node 3**  **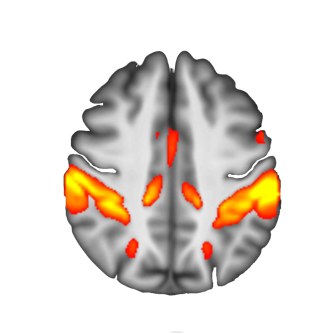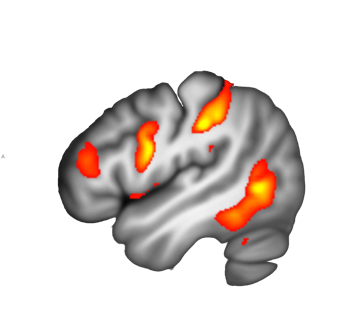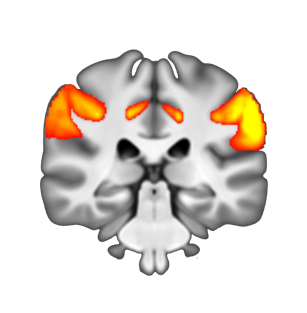** | 44% Postcentral Gyrus, 31% Anterior Supramarginal  25% Posterior Supramarginal gyrus, 8% Superior Parietal lobule  42% Posterior Cingulate, 13% Precentral | -59, -22, 32  38, -37, 39  14, -28, 39 | Pericentral Network |
| **Node 4**  **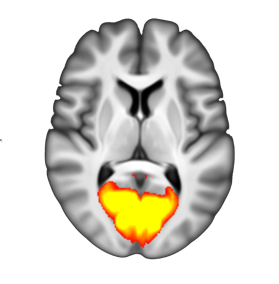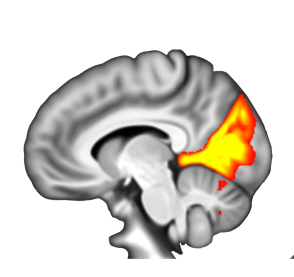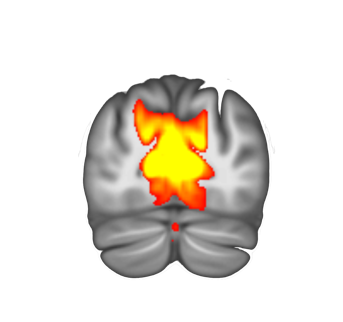** | 39% Intracalcarine Cortex, 30% Lingual gyrus  31% Cingulate, 4% Hippocampus  25% Precuneus, 17% Intracalcarine Cortex | 7, -69, 6  -5, -44, 6  25, -57, 6 | Medial Occipital Network |
| **Node 5**  **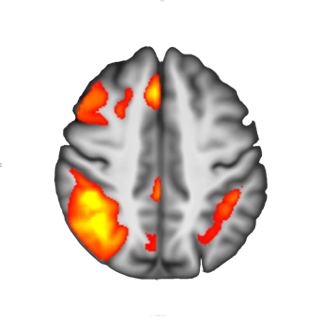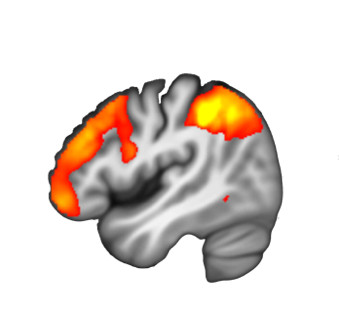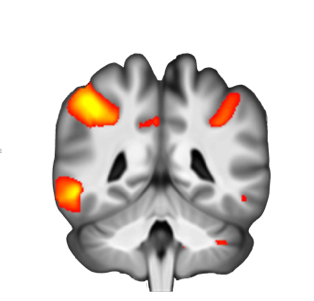** | 41% Posterior Supramarginal Gyrus, 16% Angular Gyrus  45% Paracingulate, 40% superior frontal  47% Frontal Orbital cortex, 28% frontal pole | 45, -41, 45  5, 28, 45  56, -44, -11 | Right Lateral Frontoparietal Network |
| **Node 6**  **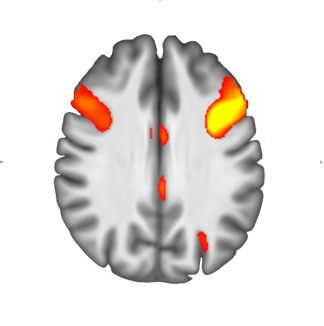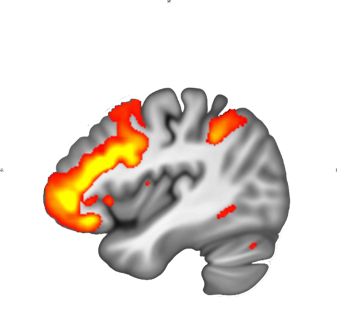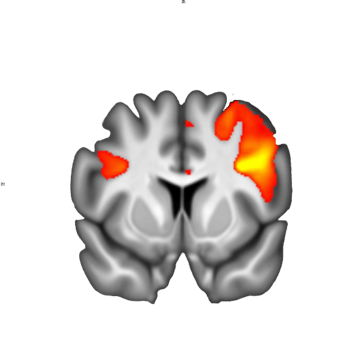** | 56% Superior Frontal Gyrus, 30% Paracingulate  25% Superior Parietal Lobule, 21% Angular Gyrus  47% Frontal Orbital cortex, 28% frontal pole | -4, 33, 42  -34, -54, 39  -37, 33, -13 | Left Lateral Frontoparietal Network |
| **Node 7**  **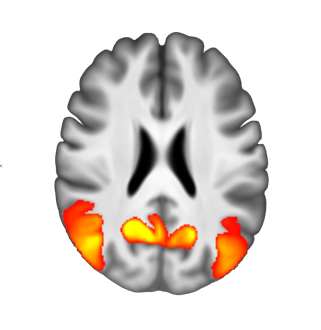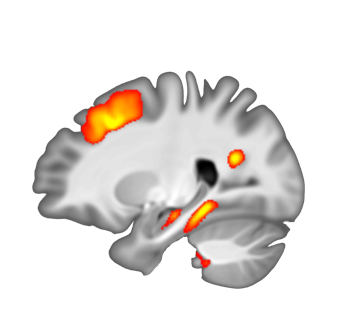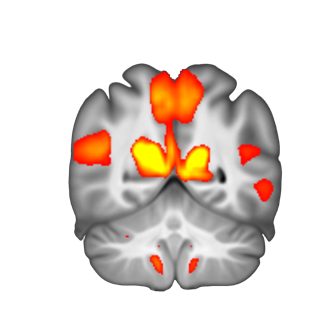** | 82% Superior Lateral Occipital Cortex  43% Precuneus, 8% Supramarginal  24, 23, 44 33% Superior Frontal, 23% Middle Frontal | -40, -80, 33  -16, -57, 15  24, 23, 44 | Medial Frontoparietal Network |
| **Node 8**  **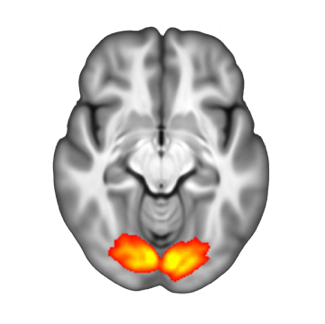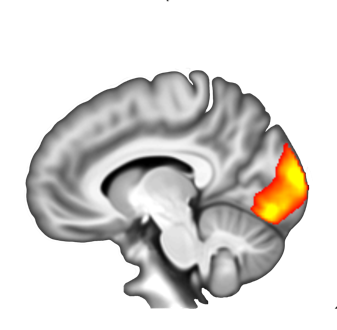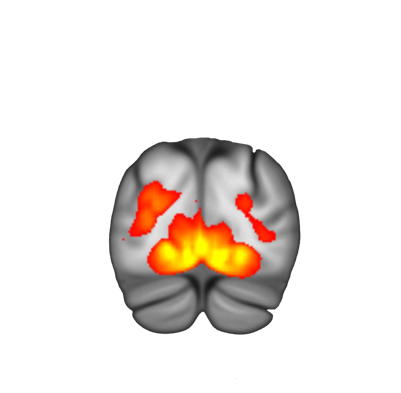** | 53% Lingual Gyrus, 11% Occipital Fusiform  62% Occipital Pole  32% Occipital Fusiform, 17% Lingual | -8, -85, -9  -8, -100, 10  18, -86, -6 | Medial Occipital Network |
| **Node 9**  **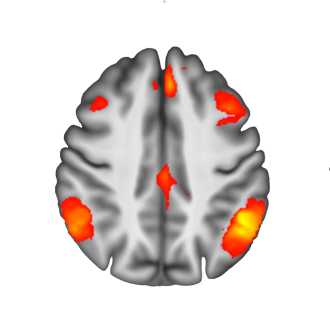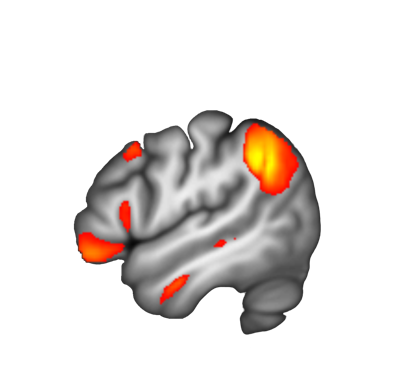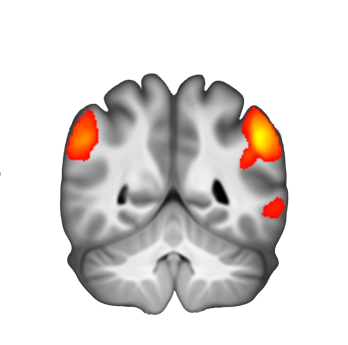** | 32% Supramarginal posterior, 19% Angular  91% Frontal Pole  63% Central Opercular cortex, 11% Parietal Operculum Cortex | -51,-50, 40  -46, 43, -13  -51, -20, 18 | Lateral Frontoparietal |
| **Node 10**  **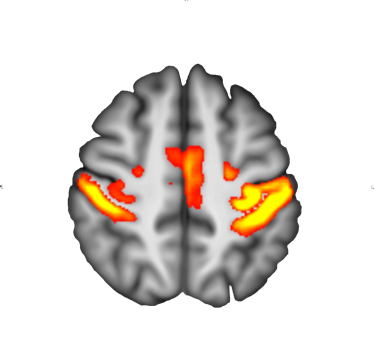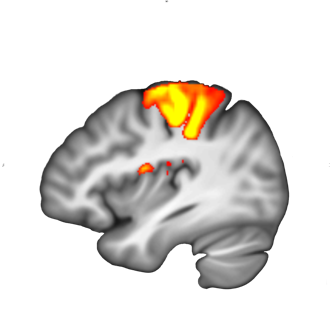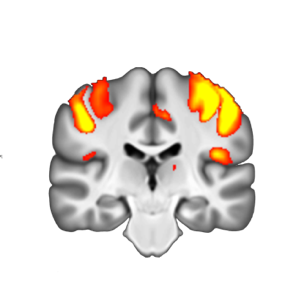** | 34% Postcentral Gyrus, 20% Supramarginal gyrus anterior  39% Postcentral, 19% Precentral Gyrus  63% Central Opercular cortex, 11% Parietal Operculum Cortex | -42, -30, 41  -35, -28, 56  -51, -20, 18 | Pericentral Network (sensorimotor) |
| **Node 11**  **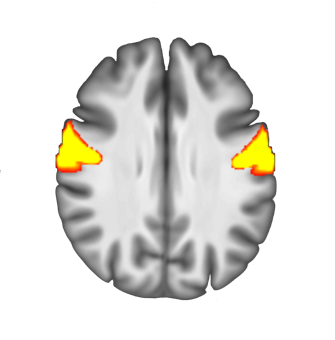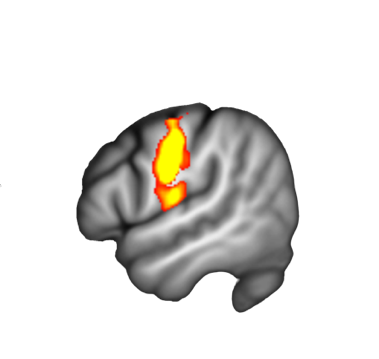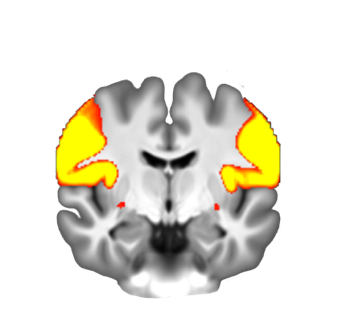** | -45% Precentral Gyrus, 33% Postcentral Gyrus  77% Central Opercular Cortex, 5% Parietal Operculum Cortex  81% Central Opercular Cortex | -52, -13, 46  -39, -13, 18  44, -7, 13 | Pericentral Network (auditory) |
| **Node 12**  **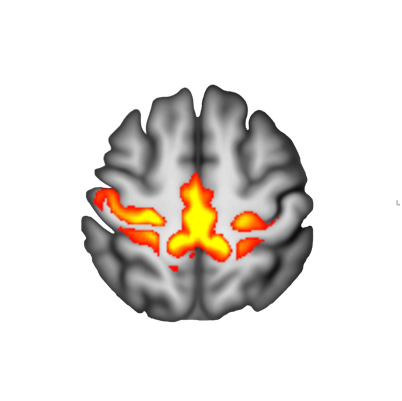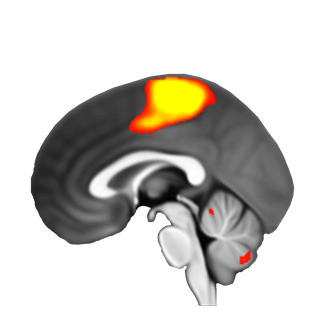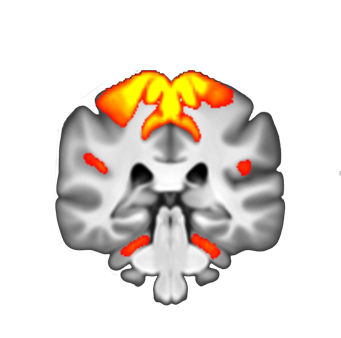** | 57% Precentral gyrus  37% Postcentral, 12% Precuneus  47% Precentral Gyrus | -6, -25, 58  -2, -44, 65  -6, -22, 64 | Pericentral Network (sensorimotor) |
| **Node 13**  **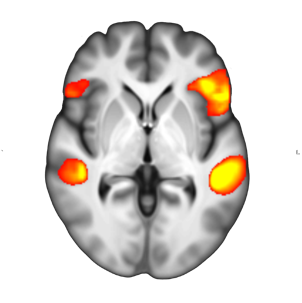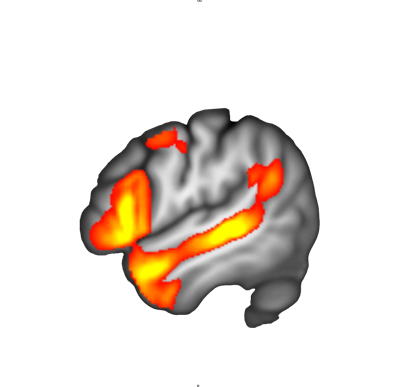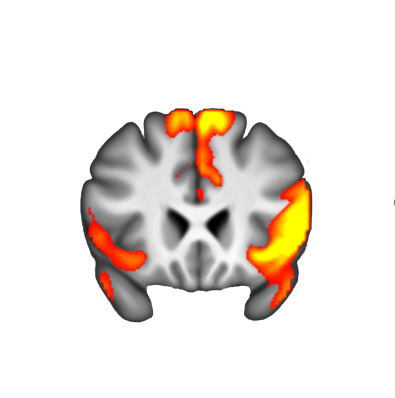** | 52% Superior Frontal Gyrus  31% Inferior frontal pars triangularis, 31% Inferior frontal pars opercularis  41% Middle Temporal posterior, 6% Superior Temporal Posterior | -11, 15, 64  -52, 21, 11  -50, -36, -3 | Mid-Cingulo Insular/ Medial Frontoparietal |
| **Node 14**  **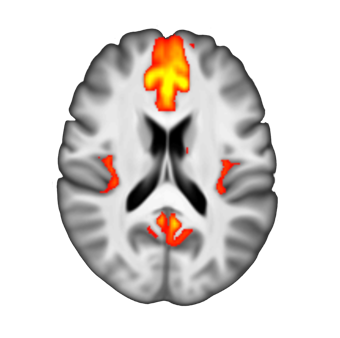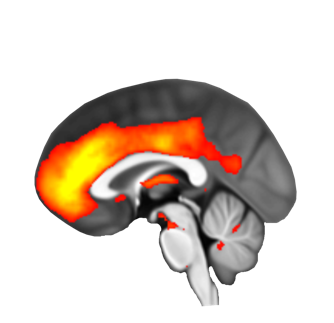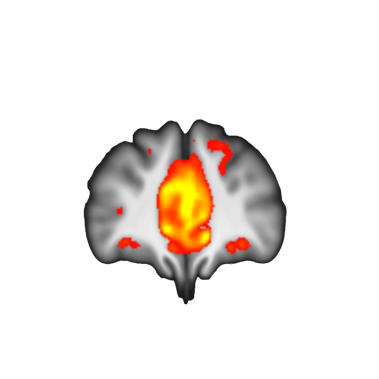** | 44% Frontal Pole, 29% Paracingulate  53% Cingulate posterior, 39% Precuneous  71% Cingulate posterior, 25% Cingulate anterior | -7, 56, 2  -4, -52, 15  2, -17, 32 | Anterior Medial Fronto-Parietal |
| **Node 15**  **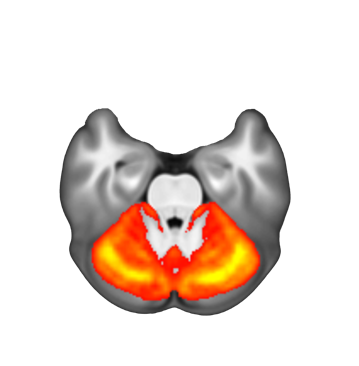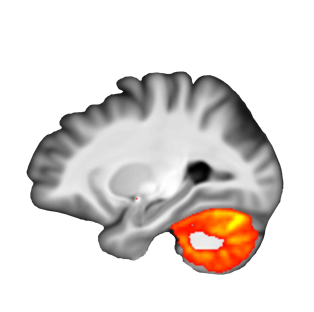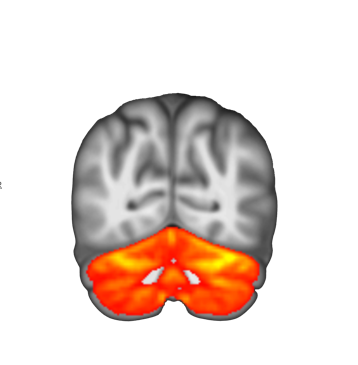** | 53.5% Cerebellum Left Crus I, 44.6% Cerebellum Left VI  62% Cerebellum Right Crus I, 33% Cerebellum Right VI | -34, -67, -24  37, -64, -24 | Cerebellar Network |
| **Node 16**  **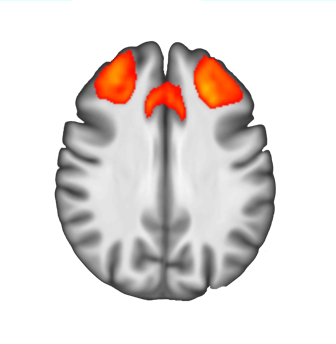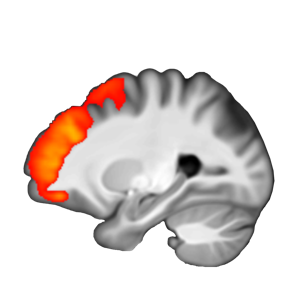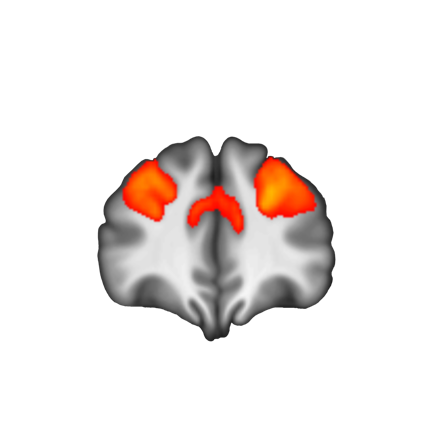** | 72% Frontal Pole  58% Paracingulate, 32% Anterior Cingulate  54% Frontal Operculum, 23% insular cortex | -27, 51, 12  5, 28, 34  -32, 18, 10 | Lateral Fronto-Parietal Network |
| **Node 17**  **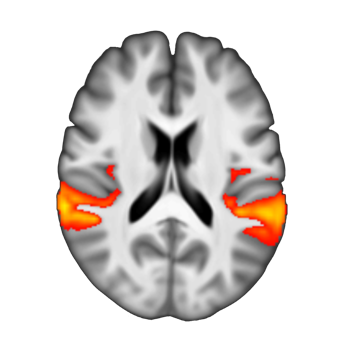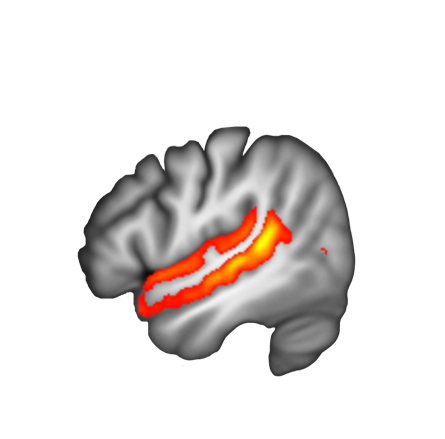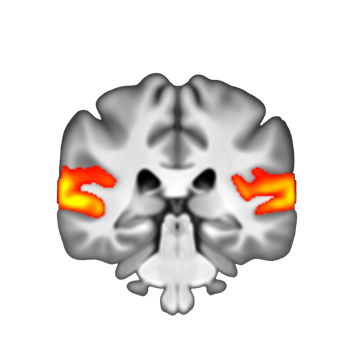** | 21% Supramarginal gyrus posterior, 14% Superior temporal gyrus posterior  34% Superior Temporal posterior, 9% Middle temporal posterior  42% Planum Polare, 19% Heschl's Gyrus | 59, -36, 7  59, -21, -2  -41, -21, -1 | Pericentral Network (auditory) |
| **Node 18**  **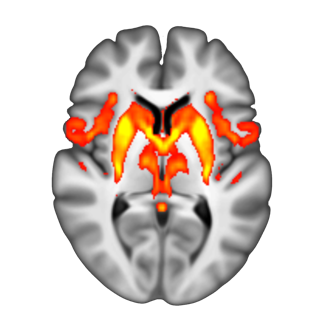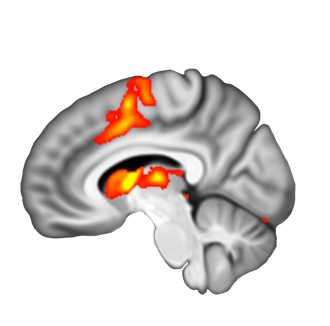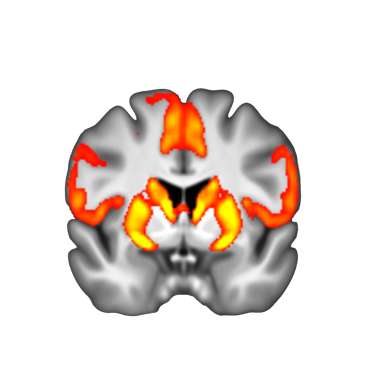** | 100% Left Putamen  41.9% Left Caudate, 38.4% Left Thalamus | -24, 7, 2  -12, -6, 16 | Basal Ganglia Network |
| **Node 19**  **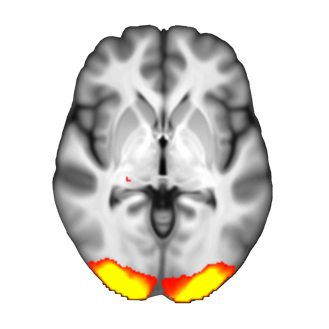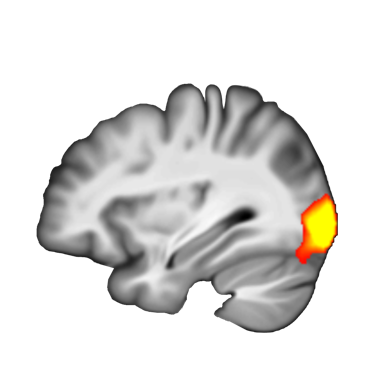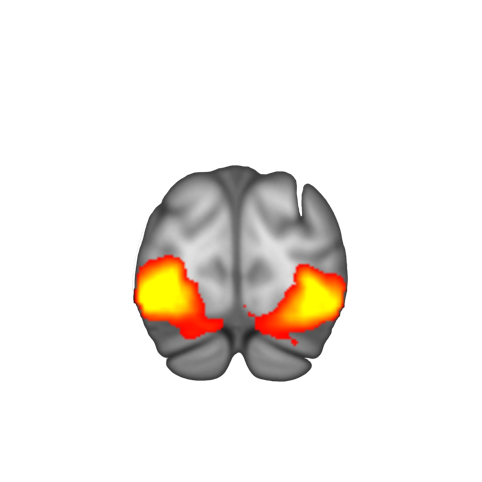** | 58% Occipital Pole, 4% Lateral Occipital Cortex Inferior  62% Occipital Pole  70% Lateral Occipital Cortex Inferior, 3% Occipital Fusiform | -23, -98, - 8  25, -99, 8  45, -81, -10 | Lateral Occipital Network |
| **Node 20**  **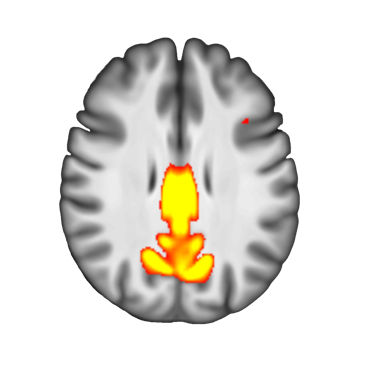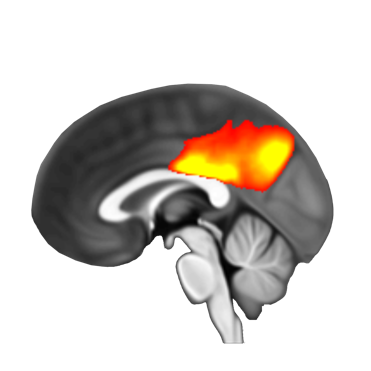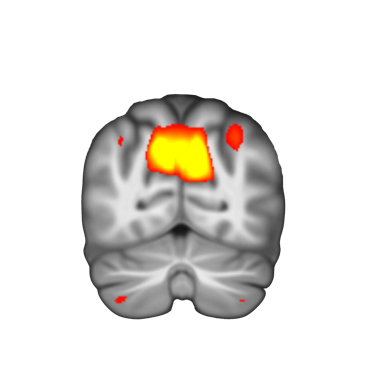** | 51% Precuneus cortex, 10% Cuneal Cortex  61% Cingulate gyrus posterior, 16% Precuneus  36% Posterior Cingulate | 14, -65, 33  -12, -48, 33  -6, -28, 27 | Medial Fronto-Parietal Network (only cingulate/precuneus) |
| **Node 21**  **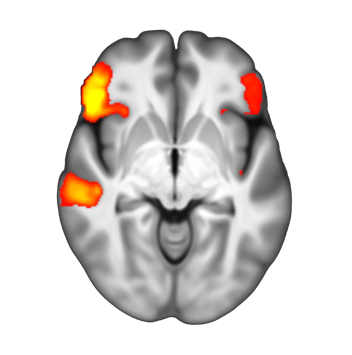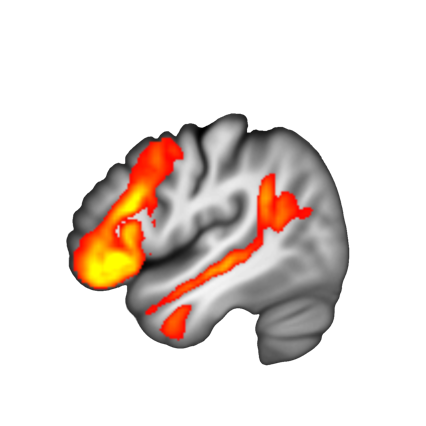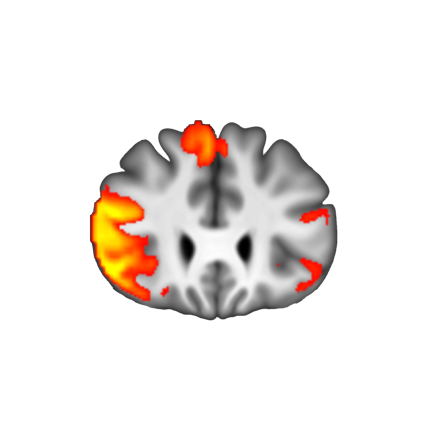** | 40% Inferior Frontal Gyrus pars triangularis, 25% Frontal orbital cortex  60% Middle Temporal posterior, 10% Superior temporal posterior  63% Superior Frontal, 25% Paracingulate | 51, 29, -6  49, -24, -8  4, 38, 40 | Right Medial Fronto-Parietal Network (without cingulate/precuneus) |

**Note:** Images were derived from the Uk Biobank Papaya Component Viewer. Reproduced by kind permission of UK Biobank ©

### **Table S2**. Highly Correlated Pairs of IDPs (R > 0.80) and Actions Taken to Reduce Multicollinearity.

| **Measurement type** | **First IDP** | **Second IDP** | | **Correlation value (>0.80)** | **Action** |
| --- | --- | --- | --- | --- | --- |
| **ASEG Subcortical Volumes** | 26596 (R ventral diencephalon)  26565 (L ventral diencephalon)  26596 (R ventral diencephalon)  26588 (R cerebellum)  26553 (L cerebral WM)  26583 (R cortex)  26562 (L hippocampus)  26585 (R lateral ventricle)  26561 (L pallidum)  26591 (R putamen)  26558 (L thalamus)  26559 (L caudate) | 26565 (L ventral diencephalon)  26526 (brainstem)  26526 (brainstem)  26557 (L cerebellum)  26584 (R cerebral WM)  26552 (L cortex)  26593 (R hippocampus)  26554 (L lateral ventricle)  26592 (R pallidum)  26560 (L putamen)  26589 (R thalamus)  26590 (R caudate) | | 0.9349618  0.8340257  0.8330799  0.9306265  0.9929878  0.9288037  0.8503003  0.9110156  0.8652333  0.9278121  0.8981629  0.9129296 | Averaged bilaterally  Averaged: combined brainstem/ventral DC  Averaged bilaterally  Averaged bilaterally  Averaged bilaterally  Averaged bilaterally  Averaged bilaterally  Averaged bilaterally  Averaged bilaterally  Averaged bilaterally  Averaged bilaterally |
| **ASEG Subcortical Intensities** | 26579 (R accumbens)  26574 (R caudate)  26541 (L cerebellum)  26575 (R putamen)  26542 (L thalamus)  26580 (R ventral DC)  26569 (R lateral ventricle) | 26548 (L accumbens)  26543 (L caudate)  26572 (R cerebellum)  26544 (L putamen)  26573 (R thalamus)  26549 (L ventral DC)  26538 (L lateral ventricle) | | 0.8666761  0.828421  0.8745482  0.8679296  0.8968732  0.8177102  0.9255214 | Averaged bilaterally  Averaged bilaterally  Averaged bilaterally  Averaged bilaterally  Averaged bilaterally  Averaged bilaterally  Averaged bilaterally |
| **Freesurfer a2009s Thickness** | 27418 (L superior frontal gyrus)  27640 (R superior frontal gyrus) | 27417 (L middle frontal gyrus)  27639 (R middle frontal gyrus) | | 0.8057944  0.8072722 | Averaged: combined lateralized variable  Averaged: combined lateralized variable |
|  |  |  | |  |  |
| **Freesurfer a2009s Volume** | No regions highly correlated >0.80 | | | | |
| **Freesurfer a2009s Area** | 27594 (R calcarine sulcus) | 27372 (L calcarine sulcus) | | 0.8080564 | Averaged bilaterally |
| **Diffusion – Probabilistic Tractography Fractional Anisotropy** | 25491 (R anterior thalamic radiation)  25501 (R inferior fronto-occipital fasciculus)  25503 (R inferior longitudinal fasciculus)  25502 (L inferior longitudinal fasciculus)  25503 (R inferior longitudinal fasciculus)  25510 (R superior longitudinal fasciculus)  25512 (R superior thalamic radiation) | 25490 (L anterior thalamic radiation)  25500 (L inferior fronto-occipital fasciculus)  25502 (L inferior longitudinal fasciculus)  25500 (L inferior fronto-occipital fasciculus)  25501 (R inferior fronto-occipital fasciculus)  25509 (L superior longitudinal fasciculus)  25111 (L superior thalamic radiation) | | 0.8704338  0.871477  0.8288225  0.8464379  0.8052722  0.8291352  0.8693731 | Averaged bilaterally  Averaged bilaterally & combined with bilateral inferior longitudinal fasciculus  Averaged bilaterally & combined with bilateral inferior fronto-occipital fasciculus  Averaged: combined lateralized variable  Averaged: combined lateralized variable  Averaged bilaterally  Averaged bilaterally |
| **Diffusion – TBSS Fractional Anisotropy** | 25078 (Anterior corona radiata)  25073 (L anterior internal capsule)  25058 (Genu of corpus callosum)  25091 (L cingulum cingulate)  25088 (R external capsule)  25094 (R Fornix cres+stria terminalis)  25066 (R inferior cerebellar peduncle)  25065 (L medial lemniscus)  25082 (R posterior corona radiata)  25075 (L posterior internal capsule)  25085 (L posterior thalamic radiation)  25068 (R superior cerebellar peduncle)  25081 (L superior corona radiata)  25096 (R superior longitudinal fasciculus | | 25079 (Anterior corona radiata)  25072 (R anterior internal capsule)  25059 (Body of corpus callosum)  25090 (R cingulum cingulate)  25089 (L external capsule)  25095 (L fornix cres+stria terminalis)  25067 (L inferior cerebellar peduncle)  25064 (R medial lemniscus)  25083 (L posterior corona radiata)  25074 (L posterior internal capsule)  25084 (R posterior thalamic radiation)  25069 (L superior cerebellar peduncle)  25080 (R superior corona radiata)  25097 (R superior longitudinal fasciculus) | 0.8782736  0.8461248  0.8200612  0.8122363  0.8629694  0.8221225  0.8477053  0.8676008  0.8390028  0.8122846  0.857639  0.8746293  0.8545769  0.8535549 | Averaged bilaterally  Averaged bilaterally  Averaged: combined variable  Averaged bilaterally  Averaged bilaterally  Averaged bilaterally  Averaged bilaterally  Averaged bilaterally  Averaged bilaterally  Averaged bilaterally  Averaged bilaterally  Averaged bilaterally  Averaged bilaterally  Averaged bilaterally |

***Legend:*** Actions taken to reduce multicollinearity between IDPs correlated with r >0.80 before being inputted into Recursive Feature Elimination analyses

### **Supplementary Results**

### **Table S3.** UK Biobank T1 Structural and Diffusion-Weighted MRI-derived IDPs Corresponding to Literature-Identified Regions.

| **Destrieux a2009s Cortical Volume IDPs** | | | | | |
| --- | --- | --- | --- | --- | --- |
| **Region Identified** | **Reference** | **Corresponding Uk Biobank IDP** | | | |
| Orbitofrontal cortex | (Blumen & Verghese, 2019; Lewis et al., 2011) | 27489 - L Inferior Frontal- Orbital Gyrus  27711 – R Inferior Frontal-Orbital Gyrus  27500 – L Orbital Gyrus  27722 – R Orbital Gyrus | | | |
| Parahippocampal gyrus/Entorhinal cortex | (Blumen & Verghese, 2019; Kanai et al., 2012; Kieckhaefer et al., 2023) | 27721– L Occipital-Temp-Med-Parahippocampal Gyrus  27499 – R Occipital-Temp-Med-Parahippocampal Gyrus | | | |
| Pre-cuneus | (Blumen & Verghese, 2019; Noonan et al., 2018) | 27506 – L Precuneus Gyrus  27728 – R Precuneus Gyrus | | | |
| Pre-central gyrus | (Blumen & Verghese, 2019) | 27505 – L Pre-central Gyrus  27727 – R Pre-central Gyrus | | | |
| Insula | (Blumen & Verghese, 2019) | 27494 – L Short Insular Gyrus  27716 – R Short Insular Gyrus | | | |
| Cingulate gyrus | (Blumen & Verghese, 2019; Kieckhaefer et al., 2023; Noonan et al., 2018) | 27482 – L Anterior Cingulate G+S  27704 – R Anterior Cingulate G+S  27483 – L Mid-Anterior Cingulate G+S  27705 – R Mid-Anterior Cingulate G+S  27484 – L Mid-Posterior Cingulate G+S  27706 – R Mid-Posterior Cingulate G+S  27485 – L Posterior-Dorsal Cingulate Gyrus | | | 27707 – R Posterior-Dorsal Cingulate Gyrus  27486 – L Posterior-Ventral Cingulate Gyrus  27708 – R Posterior-Ventral Cingulate Gyrus |
| Temporal pole | (Noonan et al., 2018) | 27519 – L Temporal pole  27741 – R Temporal pole | | | |
| Occipital pole | (Anatürk et al., 2021) | 27518 – L Occipital pole  27740 – R Occipital Pole | | | |
| Middle temporal gyrus | (Kanai et al., 2012; Kieckhaefer et al., 2023) | 27514 – L Middle Temporal Gyrus  27736 – R Middle Temporal Gyrus | | | |
| Fusiform gyrus | (Kieckhaefer et al., 2023) | 27497 – L Occipital Temporal Lateral Fusiform gyrus  27719 – R Occipital Temporal Lateral Fusiform gyrus | | | |
| Pre-frontal cortex/superior frontal/inferior frontal/middle frontal gyri | (Blumen & Verghese, 2019; Kieckhaefer et al., 2023; Kwak et al., 2018; Lewis et al., 2011; Noonan et al., 2018; Veerareddy et al., 2023) | 27488 – L Inferior Frontal Opercular Gyrus  27710 – R Inferior Frontal Opercular Gyrus  27490 – L Inferior Frontal Triangular Gyrus  27712 – R Inferior Frontal Triangular Gyrus | | | 27491 – L Middle Frontal Gyrus  27713 – R Middle Frontal Gyrus  27492 – L Superior Frontal Gyrus  27714 – R Superior Frontal Gyrus |
| **ASEG Subcortical Volume IDPs** | | | | | |
| **Region Identified** | **Reference** | **Corresponding Uk Biobank IDP** | | | |
| Hippocampus | (Blumen & Verghese, 2019) | 26562 – L Hippocampus  26593 – R Hippocampus | | | |
| Amygdala | (Blumen & Verghese, 2019; Kanai et al., 2012; Noonan et al., 2018) | 26563 – L Amygdala  26594 – R Amygdala | | | |
| Pallidum | (Blumen & Verghese, 2019) | 26561 – L Pallidum  26592 – R Pallidum | | | |
| Thalamus | (Blumen & Verghese, 2019) | 26558 – L Thalamus-Proper  26589 – R Thalamus-Proper | | | |
| Cerebellum | (Blumen & Verghese, 2019) | 26557 – L Cerebellum Cortex  26588 – R Cerebellum Cortex | | | |
| Brainstem | (Blumen & Verghese, 2019) | 26526 - Brainstem | | | |
| **Diffusion-Weighted Fractional Anisotropy IDPs** | | | | | |
| **Region Identified** | **Reference** | **Corresponding Uk Biobank IDP** | | | |
|  |  | **Probabilistic Tractography (Weighted-Mean FA)** | | **TBSS (Mean FA)** | |
| Corpus callosum | Noonan et al., 2018/ Molesworth et al.) | NA | 25059 –Body of Corpus Callosum  25058 – Genu of Corpus Callosum  25060 – Splenium of Corpus Callosum | | |
| Cingulum | (Noonan et al., 2018) | 25492 – L Cingulum Cingulate  25493 – R Cingulum Cingulate  25494 – L Cingulum Parahippocampal  25495 – R Cingulum Parahippocampal | 25091 – L Cingulum Cingulate  25090 – R Cingulum Cingulate  25093 – L Cingulum Hippocampus  25092 – R Cingulum Hippocampus | | |
| Arcuate fasciculus/ Superior longitudinal fasciculus | (Noonan et al., 2018) | 25509 – L Superior Longitudinal Fasciculus  25510 – R Superior Longitudinal Fasciculus | 25097 – L Superior Longitudinal Fasciculus  25096 – R Superior Longitudinal Fasciculus | | |
| Inferior extreme capsule/ fronto-occipital fascicule | (Noonan et al., 2018) | 25500 – L Inferior Fronto-Occipital Fasciculus  25501- R Inferior Fronto-Occipital Fasciculus | NA | | |
| Inferior longitudinal fasciculus | (Noonan et al., 2018) | 25502 – L Inferior Longitudinal Fasciculus  25503 – R Inferior Longitudinal Fasciculus | NA | | |

*Legend:* Structural grey and white matter in the UK Biobank database that were selected for the literature-based feature selection based on their correspondence with regions associated with social phenotypes in previous research

### **Table S4.** UK Biobank Resting-State fMRI IDPs corresponding to literature-selected IDPs

| **Literature** | **Nodes** | **Corresponding IDP/Edge** |
| --- | --- | --- |
| **Default Mode Network**  **(Medial Fronto-Parietal) -> Pericentral (sensorimotor)**  **(Kieckhaeefer et al., 2023)**  **30 IDPs** | 1 & 10  1 & 3  1 & 11  1 & 12  1 & 17  7 & 10  7 & 3  7 & 11  7 & 12  7 & 17  13 & 10  13 & 3  13 & 11  13 & 12  13 & 17  14 & 10  14 & 3  14 & 11  14 & 12  14 & 17  20 & 10  20 & 3  20 & 11  20 & 12  20 & 17  21 & 10  21 & 3  21 & 11  21 & 12  21 & 17 | 37  2  46  56  121  43  18  52  62  127  76  69  77  78  133  88  81  89  90  134  181  174  182  183  188  200  193  201  202  207 |
| **Lateral Fronto-parietal -> Pericentral**  **(Kieckhaeefer et al., 2023)**  **20 IDPs** | 5 & 10  5 & 3  5 & 11  5 & 12  5 & 17  6 & 10  6 & 3  6 & 11  6 & 12  6 & 17  9 & 10  9 & 3  9 & 11  9 & 12  9 & 17  16 & 10  16 & 3  16 & 11  16 & 12  16 & 17 | 41  9  50  60  125  42  13  51  61  126  45  31  54  64  129  115  108  116  117  136 |
| **Lateral Occipital -> Pericentral**  **(Anaturk et al., 2021)**  **10 IDPs** | 3 & 2  3 & 19  10 & 2  10 & 19  11 & 2  11 & 19  12 & 2  12 & 19  17 & 2  17 & 19 | 3  156  38  163  47  164  57  165  122  170 |
| **Cerebellar -> Pericentral**  **(Anaturk et al., 2021)**  **5 IDPs** | 3 & 15  10 & 15  11 & 15  12 & 15  17 & 15 | 94  101  102  103  135 |
| **Pericentral**  **(Pillemer et al., 2017)**  **10 IDPs** | 3 &10  3 & 11  3 & 12  3 & 17  10 & 11  10 & 12  10 & 17  11 & 12  11 & 17  12 & 17 | 39  48  58  123  55  65  130  66  131  132 |
| **Visual**  **(Pillemer et al., 2017)**  **6 IDPs** | 2 & 4  2 & 8  2 & 19  4 & 8  4 & 19  8 & 19 | 5  23  155  25  157  161 |

*Legend:* Resting-State fMRI IDPs in the UK Biobank database that were selected for the literature-based feature selection based on their correspondence with regions associated with social phenotypes in previous research.

**Table S6.** T1-weighted MRI-derived Structural Cortical Volume, Thickness and Area IDPs selected by RFE.

| **A2009s** | **Cortical Volume** | | **Cortical Thickness** | | **Cortical Area** | |
| --- | --- | --- | --- | --- | --- | --- |
| **Selected** | 29 (of 148) | | 43 (of 147) | | 6 (of 147) | |
|  | **Left** | **Right** | **Left** | **Right** | **Left** | **Right** |
| **Gyri** | L occipital pole*  L orbital *  L triangular portion of inferior frontal*  L inferior frontal orbital*  L pre-central*  L posterior-ventral cingulate*  L supramarginal  L medial occipito-temporal /lingual  L medial occipito-temporal/lingual  Parahippocampal*  L middle occipital  L middle temporal*  L rectus | R occipital pole*  R superior frontal*  R subcallosal | L supramarginal  L inferior frontal orbital  L posterior-ventral cingulate  L superior parietal  L occipital pole  L short insular  L anterior transverse temporal  L planum polare of the superior temporal  L planum temporalis of the superior temporal  L posterior ramus of the lateral fissure  L horizontal ramus of the anterior lateral fissure | R supramarginal  R rectus  R superior & middle frontal  R superior pre-central  R precuneus  R orbital  R planum polare of the superior temporal  R inferior temporal  R middle temporal  R temporal pole | L orbital | R orbital  R superior precentral |
| **Sulci** | L central  L orbital  L lateral orbital  L postcentral  L pericallosal  L posterior transverse collateral | R superior temporal  R inferior temporal  R superior frontal  R posterior lateral fissure  R superior pre-central  R marginal | L central  L middle frontal  L inferior frontal  L lateral occipito-temporal  L temporal transverse  L intraparietal/transverse parietal  L superior circular insular  L inferior circular insular | R post-central  R middle frontal  R orbital  R superior frontal  R calcarine  R inferior temporal  R inferior circular insular | L central  L orbital  L lateral orbital  L orbital | R orbital |
| **Gyri + Sulci** | L frontomarginal G+S  L long insular G+S |  | L paracentral G+S  L mid-anterior cingulate G+S  L anterior cingulate G+S  L mid-posterior cingulate  L inferior occipital G+S | R paracentral G+S  R subcentral G+S  G+S |  |  |

Legend: Cortical volume, thickness and area IDPs selected by the RFE as associated with social participation. *Indicates brain regions implicated in previous literature. L left hemisphere, R right hemisphere, G+S: where Freesurfer derives both as a single measure.

### **Table S7.** Structural Subcortical Volume and Intensity IDPs Selected by Recursive Feature Elimination

| **ASEG** | **ASEG Subcortical Volume** | **ASEG Subcortical Intensity** |
| --- | --- | --- |
| **Number Selected** | 11 (of 31) | 31 (of 31) |
| **Selected IDPs** | BA pallidum*  Central Corpus Callosum  Posterior Corpus Callosum  BA putamen  BA caudate  BA lateral ventricles  L amygdala*  BA thalamus*  BA Cerebral WM  L Cerebellum WM*  BA Cortex | All IDPs selected |

*Legend:* Subcortical volume and intensity IDPs selected by the RFE as important for social participation. *Indicates regions implicated in previous literature. L left hemisphere, R right hemisphere, BA bilateral averaged.

**Table S8.** Diffusion-Weighted Fractional Anisotropy IDPs Selected by Recursive Feature Elimination

| **Diffusion** | **TBSS – Mean FA** | **Probabilistic Tractography – Weighted-mean FA** |
| --- | --- | --- |
| **Number selected** | 9 (of 34) | 20 (of 21) |
| **Selected IDPs** | BA superior longitudinal fasciculus*  BA Superior cerebellar peduncle  BA posterior internal capsule  L sagittal striatum  R cerebral peduncle  Middle cerebellar peduncle  BA cingulum cingulate part*  BA Anterior corona radiata  L cingulum hippocampal part* | BA Superior Thalamic Radiation  BA Anterior Thalamic Radiation  BA Superior Longitudinal Fasciculus*  R Posterior Thalamic Radiation  L Posterior Thalamic Radiation  L Medial Lemniscus  Forceps Minor  R Corticospinal  R Uncinate Fasciculus  L Uncinate Fasciculus  R Medial Lemniscus  Middle Cerebellar Peduncle  Forceps Major  L Corticospinal  L Cingulum Parahippocampal part*  L Cingulum Cingulate part*  R Acoustic Radiation  L Acoustic Radiation  R Cingulum Parahippocampal part*  BA Inferior longitudinal/fronto-occipital fasciculi* |

*Legend*: Mean FA and Weighted-Mean FA IDPs selected by the RFE as important for social participation. *Indicates brain regions implicated in previous literature. L left hemisphere, R right hemisphere, BA bilateral averaged.

### **Table S9.** Resting-State Functional Connectivity IDPs selected by Recursive Feature Elimination.

| **Total Number of IDPs selected: 49 (of 210)** | | | |
| --- | --- | --- | --- |
| **Selected Edge** | **Nodes Involved** | **Average partial correlation between nodes** | **Networks** |
| Edge 4  Edge 6  Edge 9*  Edge 14  Edge 17  Edge 21  Edge 25*  Edge 29  Edge 30  Edge 31*  Edge 33  Edge 34  Edge 35  Edge 39*  Edge 43*  Edge 56*  Edge 58*  Edge 60*  Edge 63  Edge 67  Edge 71  Edge 72  Edge 86  Edge 88*  Edge 92  Edge 93  Edge 95  Edge 97  Edge 103*  Edge 105  Edge 115*  Edge 129*  Edge 136*  Edge 137  Edge 139  Edge 147  Edge 148  Edge 156*  Edge 162  Edge 164*  Edge 182*  Edge 183*  Edge 184  Edge 191  Edge 193*  Edge 199  Edge 200*  Edge 206  Edge 210 | 1 & 4  3 & 4  3 & 5  4 & 6  2 & 7  6 & 7  4 & 8  1 & 9  2 & 9  3 & 9  5 & 9  6 & 9  7 & 9  3 & 10  7 & 10  1 & 12  3 & 12  5 & 12  8 & 12  1 & 13  5 & 13  6 & 13  8 & 14  10 & 14  1 & 15  2 & 15  4 & 15  6 & 15  12 & 15  14 & 15  10 & 16  9 & 17  16 & 17  1 & 18  3 & 18  11 & 18  12 & 18  3 & 19  9 & 19  11 & 19  11 & 20  12 & 20  13 & 20  1 & 21  3 & 21  9 & 21  10 & 21  16 & 21  20 & 21 | -0.68  -0.70  -0.50  -0.87  -0.27  -0.09  2.78  2.50  -2.24  -1.54  0.32  1.01  -0.73  0.66  -0.70  -0.34  0.26  -0.97  -0.42  0.68  -1.36  1.04  -0.12  -0.43  -0.51  -0.62  -0.09  -0.40  -0.18  -0.23  -0.43  0.29  -0.78  -0.62  0.31  0.29  0.16  -0.87  -0.56  -0.09  -0.23  -0.26  -0.36  -0.27  0.20  1.31  -1.08  0.31  -0.47 | MFP & MOC  PC & MOC  PC & Right LFP  MOC & Left LFP  LOC & MFP  Left LFP & MFP  MOC & MOC•  MFP & LFP  LOC & LFP  PC & LFP  Right LFP & LFP•  Left LFP & LFP•  MFP & LFP  PC & PC (somatomotor)•  MFP & PC (somatomotor)  MFP & PC (somatomotor)  PC & PC (somatomotor)•  Right LFP & PC (somatomotor)  MOC & PC (somatomotor)  MFP & MCI/ MFP•  Right LFP & MCI/ MFP  Left LFP & MCI/ MFP  MOC & MFP (Anterior)  PC (somatomotor) & MFP (Anterior)  MFP & CB  LOC & CB  MOC & CB  Left LFP & CB  PC (somatomotor) & CB  MFP (Anterior) & CB  PC (somatomotor) & LFP  LFP & PC (auditory)  LFP & PC (auditory)  MFP & BG  PC & BG  PC (auditory) & BG  PC (somatomotor) & BG  PC & LOC  LFP & LOC  PC (auditory) & LOC  PC (auditory) & MFP (Posterior)  PC (somatomotor) & MFP (Posterior)  MCI/ MFP & MFP (Posterior)**•**  MFP & Right MFP•  PC & Right MFP  LFP & Right MFP  PC (somatomotor) & Right MFP  LFP & Right MFP  MFP (Posterior) & MFP• |

*Legend:* Functional connectivity (Edges) IDPs selected by the RFE as important for social participation. *Indicates IDPs implicated in previous literature. • Indicates intra-network connectivity. PC Pericentral, MFP Medial Fronto-Parietal, MOC Medial Occipital, LOC lateral occipital, LFP Lateral Fronto-Parietal, MCI Mid-Cingulo Insular, BG Basal-Ganglia, CB Cerebellar.

**References**

Alfaro-Almagro, F., Jenkinson, M., Bangerter, N. K., Andersson, J. L. R., Griffanti, L., Douaud, G., Sotiropoulos, S. N., Jbabdi, S., Hernandez-Fernandez, M., Vallee, E., Vidaurre, D., Webster, M., McCarthy, P., Rorden, C., Daducci, A., Alexander, D. C., Zhang, H., Dragonu, I., Matthews, P. M., … Smith, S. M. (2018). Image processing and Quality Control for the first 10,000 brain imaging datasets from UK Biobank. *NeuroImage*, *166*, 400–424. https://doi.org/10.1016/j.neuroimage.2017.10.034

Anatürk, M., Suri, S., Smith, S. M., Ebmeier, K. P., & Sexton, C. E. (2021). Leisure Activities and Their Relationship With MRI Measures of Brain Structure, Functional Connectivity, and Cognition in the UK Biobank Cohort. *Frontiers in Aging Neuroscience*, *13*, 734866. https://doi.org/10.3389/fnagi.2021.734866

Beckmann, C. F., DeLuca, M., Devlin, J. T., & Smith, S. M. (2005). Investigations into resting-state connectivity using independent component analysis. *Philosophical Transactions of the Royal Society B: Biological Sciences*, *360*(1457), 1001–1013. https://doi.org/10.1098/rstb.2005.1634

Bellani, M., Bontempi, P., Zovetti, N., Gloria Rossetti, M., Perlini, C., Dusi, N., Squarcina, L., Marinelli, V., Zoccatelli, G., Alessandrini, F., Francesca Maria Ciceri, E., Sbarbati, A., & Brambilla, P. (2020). Resting state networks activity in euthymic bipolar disorder. *Bipolar Disorders*, *22*(6), 593–601. https://doi.org/10.1111/bdi.12900

Blumen, H. M., & Verghese, J. (2019). Gray matter volume covariance networks associated with social networks in older adults. *Social Neuroscience*, *14*(5), 559–570. https://doi.org/10.1080/17470919.2018.1535999

Destrieux, C., Fischl, B., Dale, A., & Halgren, E. (2010). Automatic parcellation of human cortical gyri and sulci using standard anatomical nomenclature. *NeuroImage*, *53*(1), 1–15. https://doi.org/10.1016/j.neuroimage.2010.06.010

Eickhoff, S. B., Stephan, K. E., Mohlberg, H., Grefkes, C., Fink, G. R., Amunts, K., & Zilles, K. (2005). A new SPM toolbox for combining probabilistic cytoarchitectonic maps and functional imaging data. *NeuroImage*, *25*(4), 1325–1335. https://doi.org/10.1016/j.neuroimage.2004.12.034

Fischl, B. (2012). FreeSurfer. *NeuroImage*, *62*(2), 774–781. https://doi.org/10.1016/j.neuroimage.2012.01.021

Kanai, R., Bahrami, B., Roylance, R., & Rees, G. (2012). Online social network size is reflected in human brain structure. *Proceedings of the Royal Society B: Biological Sciences*, *279*(1732), 1327–1334. https://doi.org/10.1098/rspb.2011.1959

Kieckhaefer, C., Schilbach, L., & Bzdok, D. (2023). Social belonging: Brain structure and function is linked to membership in sports teams, religious groups, and social clubs. *Cerebral Cortex*, *33*(8), 4405–4420. https://doi.org/10.1093/cercor/bhac351

Kwak, S., Joo, W., Youm, Y., & Chey, J. (2018). Social brain volume is associated with in-degree social network size among older adults. *Proceedings of the Royal Society B: Biological Sciences*, *285*(1871), 20172708. https://doi.org/10.1098/rspb.2017.2708

Laird, A. R., Fox, P. M., Eickhoff, S. B., Turner, J. A., Ray, K. L., McKay, D. R., Glahn, D. C., Beckmann, C. F., Smith, S. M., & Fox, P. T. (2011). Behavioral Interpretations of Intrinsic Connectivity Networks. *Journal of Cognitive Neuroscience*, *23*(12), 4022–4037. https://doi.org/10.1162/jocn_a_00077

Lewis, P. A., Rezaie, R., Brown, R., Roberts, N., & Dunbar, R. I. M. (2011). Ventromedial prefrontal volume predicts understanding of others and social network size. *NeuroImage*, *57*(4), 1624–1629. https://doi.org/10.1016/j.neuroimage.2011.05.030

Noonan, M. P., Mars, R. B., Sallet, J., Dunbar, R. I. M., & Fellows, L. K. (2018). The structural and functional brain networks that support human social networks. *Behavioural Brain Research*, *355*, 12–23. https://doi.org/10.1016/j.bbr.2018.02.019

Okbay, A., Wu, Y., Wang, N., Jayashankar, H., Bennett, M., Nehzati, S. M., Sidorenko, J., Kweon, H., Goldman, G., Gjorgjieva, T., Jiang, Y., Hicks, B., Tian, C., Hinds, D. A., Ahlskog, R., Magnusson, P. K. E., Oskarsson, S., Hayward, C., Campbell, A., … Young, A. I. (2022). Polygenic prediction of educational attainment within and between families from genome-wide association analyses in 3 million individuals. *Nature Genetics*, *54*(4), 437–449. https://doi.org/10.1038/s41588-022-01016-z

Robinson, S., Basso, G., Soldati, N., Sailer, U., Jovicich, J., Bruzzone, L., Kryspin-Exner, I., Bauer, H., & Moser, E. (2009). A resting state network in the motor control circuit of the basal ganglia. *BMC Neuroscience*, *10*(1), 137. https://doi.org/10.1186/1471-2202-10-137

Smith, S. M., Alfaro-Almagro, F., & Miller, K. L. (2024). *UK Biobank Brain Imaging Documentation*. *1.10*.

Smith, S. M., Fox, P. T., Miller, K. L., Glahn, D. C., Fox, P. M., Mackay, C. E., Filippini, N., Watkins, K. E., Toro, R., Laird, A. R., & Beckmann, C. F. (2009). Correspondence of the brain’s functional architecture during activation and rest. *Proceedings of the National Academy of Sciences*, *106*(31), 13040–13045. https://doi.org/10.1073/pnas.0905267106

Smith, S. M., Hyvärinen, A., Varoquaux, G., Miller, K. L., & Beckmann, C. F. (2014). Group-PCA for very large fMRI datasets. *NeuroImage*, *101*, 738–749. https://doi.org/10.1016/j.neuroimage.2014.07.051

Uddin, L. Q., Yeo, B. T. T., & Spreng, R. N. (2019). Towards a Universal Taxonomy of Macro-scale Functional Human Brain Networks. *Brain Topography*, *32*(6), 926–942. https://doi.org/10.1007/s10548-019-00744-6

Veerareddy, A., Fang, H., Safari, N., Xu, P., & Krueger, F. (2023). Cognitive empathy mediates the relationship between gray matter volume size of dorsomedial prefrontal cortex and social network size: A voxel-based morphometry study. *Cortex*, *169*, 279–289. https://doi.org/10.1016/j.cortex.2023.09.015
